# The Impact of COVID-19 on the Cardiovascular Health of Emerging Adults Aged 18-25: Findings From a Scoping Review

**DOI:** 10.1016/j.cjcpc.2022.11.005

**Published:** 2022-12-01

**Authors:** Zachary V. Rezler, Emma Ko, Elaine Jin, Misha Ishtiaq, Christina Papaioannou, Helena Kim, Kyobin Hwang, Yu-Hsin (Sophy) Lin, Jake Colautti, Karen M. Davison, Vidhi Thakkar

**Affiliations:** a Bachelor of Health Sciences (Honours) Program, Faculty of Health Sciences, McMaster University, Hamilton, Ontario, Canada; b Undergraduate Medical Education Program, Faculty of Health Sciences, Michael G. DeGroote School of Medicine, McMaster University, Hamilton, Ontario, Canada; c Health Science Program, Faculty of Science and Horticulture, Kwantlen Polytechnic University, Surrey, British Columbia, Canada

## Abstract

There is limited knowledge regarding the cardiovascular impact of coronavirus disease 2019 (COVID-19) on emerging adults aged 18-25, a group that disproportionately contracts COVID-19. To guide future cardiovascular disease (CVD) research, policy, and practice, a scoping review was conducted to: (i) examine the impact of the COVID-19 pandemic on the cardiovascular health of emerging adults; and (ii) identify strategies to screen for and manage COVID-19–related cardiovascular complications in this age group. A comprehensive search strategy was applied to several academic databases and grey literature sources. An updated search yielded 6738 articles, 147 of which were extracted and synthesized. Reports identified COVID-19–associated cardiac abnormalities, vascular alterations, and multisystem inflammatory syndrome in emerging adults; based on data from student-athlete samples, prevalence estimates of myocarditis and cardiac abnormalities were 0.5%-3% and 0%-7%, respectively. Obesity, hypertension, CVD, congenital heart disease, and marginalization are potential risk factors for severe COVID-19, related cardiovascular complications, and mortality in this age group. As a screening modality for COVID-19–associated cardiac involvement, it is recommended that cardiac magnetic resonance imaging be indicated by a positive cardiac history and/or abnormal “triad” testing (cardiac troponin, electrocardiogram, and transthoracic echocardiogram) to improve diagnostic utility. To foster long-term cardiovascular health among emerging adults, cardiorespiratory fitness, health literacy and education, and telehealth accessibility should be priorities of health policy and clinical practice. Ultimately, surveillance data from the broader emerging adult population will be crucial to assess the long-term cardiovascular impact of both COVID-19 infection and vaccination, guide screening and management protocols, and inform CVD prevention efforts.

## Résumé

Il existe peu de données portant sur les répercussions de la maladie à coronavirus 2019 (COVID-19) sur le plan cardiovasculaire chez les jeunes adultes âgés de 18 à 25 ans, un groupe contractant la COVID-19 de façon disproportionnée. Afin d’orienter la recherche, les poli-tiques et les pratiques en matière de maladies cardiovasculaires (MCV), un examen exploratoire a été réalisé dans le but i) d’examiner les conséquences de la pandémie de la COVID-19 sur la santé cardiovasculaire des jeunes adultes, et ii) de proposer des stratégies de dépistage et de prise en charge des complications cardiovasculaires associées à la COVID-19 chez les personnes de cette tranche d’âge. Une recherche initiale exhaustive a été réalisée dans plusieurs bases de données universitaires et sources de littérature grise. Les résultats actualisés de cette recherche ont permis de recenser 6 738 articles, dont 147 ont été extraits et synthétisés. Les rapports faisaient état d’anomalies cardiaques, d’altérations vasculaires et de cas du syndrome inflammatoire multisystémique, tous associés à la COVID-19 chez les jeunes adultes. À la lumière des données sur les échantillons d’étudiants-athlètes, la prévalence des myocardites et des anomalies cardiaques se situait respectivement entre 0,5 et 3 %, et entre 0 et 7 % environ. Chez ce même groupe d’âge, l’obésité, l’hypertension, les MCV, les cardiopathies congénitales et la marginalisation constituent des facteurs de risque de COVID-19 sévère, de complications cardiovasculaires associées à la COVID-19 et de mortalité. Dans le cadre du dépistage des atteintes cardiaques associées à la COVID-19, il est recommandé, pour améliorer l’utilité diagnostique, d’indiquer l’imagerie par résonance magnétique cardiaque lors de l’existence d’antécédents cardiaques ou à la suite d’une « triade » de dépistages anormaux (la troponine cardiaque, l’électrocardiogramme et l’échocardiographie transthoracique). Afin de favoriser une bonne santé cardiovasculaire à long terme chez les jeunes adultes, il est recommandé que la capacité cardiorespiratoire, la littératie dans le domaine de la santé, l’éducation et l’accès à la télésanté soient intégrés à titre de priorités dans les politiques de santé et la pratique clinique. En définitive, les données de surveillance portant sur cette large tranche d’âge seront essentielles pour évaluer les répercussions cardiovasculaires à long terme (autant celles d'infections à la COVID-19 que celles de la vaccination), pour orienter les protocoles de dépistage et de prise en charge, ainsi que pour éclairer les efforts de prévention des MCV.

Coronavirus disease 2019 (COVID-19) illness severity varies among those infected, and though the majority of individuals present with a mild cough and flu-like symptoms, others may experience pneumonia, virus-related cardiac injury, and/or death.^1^ Individuals with cardiovascular comorbidities are at a greater risk of experiencing more severe COVID-19, likely due to the virus exacerbating pre-existing complications through cardiac involvement.^2^^,^^3^ However, even in otherwise healthy individuals, infection can lead to alterations in heart structure (eg, fibrosis) and function. Karbalai Saleh et al.^4^ found that nearly 30% of their COVID-19 hospitalized patients (mean age = 59 years) experienced cardiac injury, which was associated with a nearly 2-fold increase in risk of short-term mortality. Moreover, there are data to suggest that individuals may be delaying or avoiding medical treatment for cardiovascular emergencies in response to the pandemic, likely resulting in worse cardiovascular outcomes and mortality. For example, cardiac centres in Canada reported a 30% reduction in emergency visits for ST-elevation myocardial infarctions earlier in 2020.^5^

### The impact of COVID-19 on the cardiovascular health of emerging adults

The majority of research on COVID-19 has focused on cardiovascular complications in vulnerable populations, such as older adults and those with pre-existing chronic conditions.^4^^,^^6^ Research specific to emerging adults (ie, those aged 18-25^7^^,^^8^) is lacking; however, there is preliminary evidence demonstrating myocarditis and cardiac abnormalities in as many as 58% of college athletes following COVID-19.^9^, ^10^, ^11^ As of February 2022, Canadians between the ages of 20 and 29 (accounting for 13% of the total population^12^) represented nearly one-fifth (n = 610,151) of COVID-19 cases and accounted for 4.8% (n = 6346), 3.1% (n = 699), and 0.3% (n = 106) of hospitalizations, intensive care unit (ICU) admissions, and deaths, respectively.^13^ Cardiovascular complications, including cardiogenic shock and arrhythmias, have been observed in COVID-19 patients 18 years old and younger.^14^ Multisystem inflammatory syndrome in children/adults (MIS-C/A) and life-threatening cardiovascular presentations (eg, myocarditis) have also been reported among young adults with or after COVID-19, the long-term consequences of which remain unknown.^15^ Furthermore, in light of global vaccination efforts, new evidence has emerged concerning the cardiovascular safety of COVID-19 vaccines (eg, vaccine-related myocarditis) in younger age groups.^16^^,^^17^ Because emerging adults disproportionately contract COVID-19,^18^^,^^19^ determining the short- and long-term impacts of infection on cardiovascular function and cardiovascular disease (CVD) risk will be of value to clinicians and policymakers and better inform COVID-19 vaccination policy.

### Objectives

The current knowledge gaps surrounding the impact of COVID-19 on the cardiovascular health of emerging adults relate to: (i) the prevalence of COVID-19–related cardiovascular presentations/complications; (ii) the appropriate screening and management of such conditions; and (iii) cardiovascular care. Therefore, to address the needs of various knowledge users (eg, policymakers, programme planners, and health care providers), a scoping review was conducted to: (i) describe the impact of the COVID-19 pandemic on the cardiovascular health of emerging adults; and (ii) identify strategies to screen for and manage cardiovascular complications in emerging adults.

## Methods

The Preferred Reporting Items for Systematic Reviews and Meta-Analyses Extension for Scoping Reviews (PRISMA-ScR)^20^ guided this scoping review. Scoping reviews are conducted to examine the extent, range, and nature of the evidence surrounding a given topic and often precede systematic reviews when evidence in an area is new or limited. These reviews map findings from a broader evidence set and identify current gaps in the literature to support future research.^20^ In comparison, systematic reviews additionally appraise the literature (eg, assess risk of bias) and are more appropriate when there are clearly defined research questions.

### Search strategy

In consultation with a research librarian from McMaster University, the search strategy was developed and applied to the following bibliographic databases: MEDLINE (via Ovid), Cumulative Index to Nursing and Allied Health Literature (CINAHL), Embase, Web of Science, PsycInfo (via ProQuest), and Sociological Abstracts. All identified keywords and index terms related to COVID-19, emerging adults, and the cardiovascular conditions of interest were included in the search and adapted for each database (Supplemental Table S1). Bibliographic databases were initially searched on January 22, 2021, and updated on January 16, 2022; no date limits were applied.

Additional searches were conducted up until January 16, 2022, to locate grey literature from national and international sources, including the Cardiac Health Foundation of Canada, Heart and Stroke Foundation of Canada, Hypertension Canada, McMaster Health Forum, and World Health Organization.

### Inclusion criteria

English-language articles were required to include the following: (i) emerging adults (individuals aged approximately 18-25 ± 2) as the main participant group or subgroup, or postsecondary student samples with a mean age between 18 and 25; (ii) a COVID-19 context; and (iii) at least one of the cardiovascular conditions of interest (Box 1). The age range defining emerging adults varies across the literature, often focusing on 18- to 25-year-olds but occasionally spanning 18-29.^7^^,^^8^ The former definition (ie, ages 18-25) was selected by the research team; however, when applicable, 18- to 29-year-old cohorts were included in the review. All study designs were considered for inclusion.

**Box 1 Cardiovascular conditions included in the screening criteria**

• Hypertension or high blood pressure
• Arrhythmia (includes tachycardia, bradycardia, atrial fibrillation)
• Myocardial infarction
• Heart failure
• Cardiac arrest
• Coronary heart disease or ischemic heart disease
• Stroke or cerebrovascular accident
• Transient ischemic attack
• Valvular heart disease
• Cardiomyopathy (includes myocarditis)
• Cardiovascular abnormalities

### Exclusion criteria

Studies were excluded if they: (i) focused on emerging adults who were pregnant or studying/working in health care; (ii) did not reference COVID-19 or the cardiovascular conditions outlined in the inclusion criteria; or (iii) were not available in full text, with the exception of case reports and case series. Articles related to COVID-19 vaccines were excluded in the updated search as they were not part of the initial scope of this project; however, these articles were collected during the screening phase to provide additional context and evidence for discussion.

### Selection of studies

Citations were collated and imported into Covidence,^21^ and duplicates were automatically removed. After a pilot test of the screening protocol, each title and abstract were screened independently by 2 reviewers (MI, HK, KH, Y-HL, JC) and assessed against the established inclusion/exclusion criteria. Sources that appeared to satisfy the inclusion criteria were then retrieved as full-text articles and examined by 2 independent reviewers (MI, HK, KH, Y-HL, JC). Disagreements that occurred between reviewers were resolved by the primary author (ZVR).

### Data extraction, quality assessment, and analysis

A standardized data extraction form was completed for each included study to gather the following information: publication details (publication year and full citation), participant characteristics (country, population, and age), study characteristics (aim, design, methods, inclusion/exclusion criteria, sample size, measures, and interventions), cardiovascular health outcomes, pertinent findings (eg, risk and protective factors and prevalence and incidence estimates), conclusions, and implications. Data extracted from included articles were organized into Supplemental Tables S2 and S3. In accordance with the PRISMA-ScR,^20^ articles were not appraised for risk of bias or methodological quality. Thematic content analysis, based on the scoping review objectives, was conducted by the primary author (ZVR) with assistance from researcher assistants (EK, EJ, MI, CP) and guidance from 2 senior researchers (KMD, VT).

## Results

### Search results

**Figure 1:**
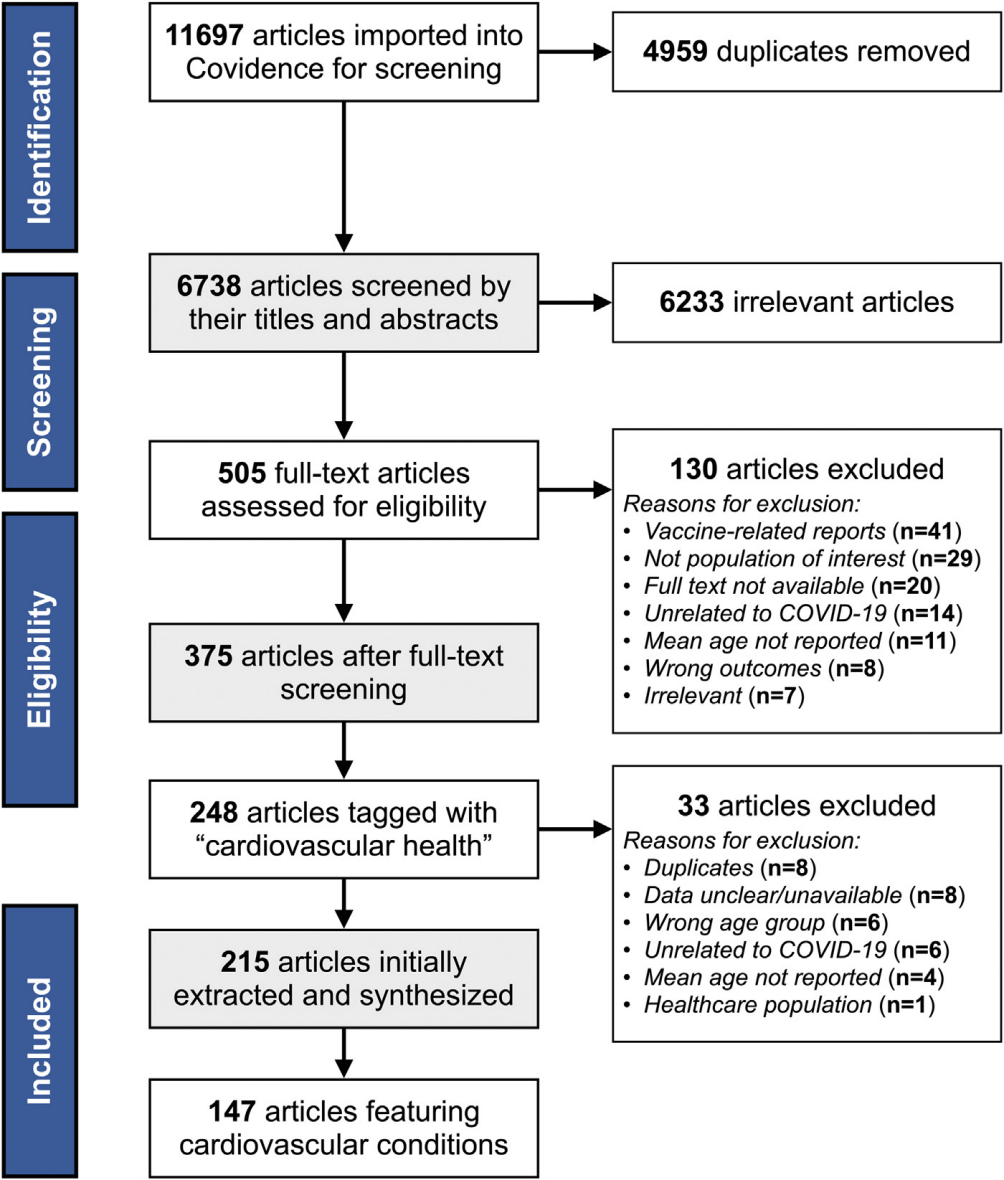
Preferred Reporting Items for Systematic Reviews and Meta-Analyses (PRISMA) flowchart of the search results.

The academic database searches yielded 6738 articles after the automatic removal of duplicates, of which 6233 were deemed irrelevant during abstract and title screening. A total of 505 articles then underwent full-text screening, of which 130 were excluded for reasons such as being COVID-19 vaccine related (n = 41), including participants outside of the established age criteria (n = 24), not being available in full text (n = 19), not being directly related to COVID-19 (n = 14), or failing to report the mean age of participants (n = 11) (see Fig. 1). In total, 248 articles captured cardiovascular health outcomes, though 33 were excluded during the data extraction phase as they were either duplicates or did not meet the inclusion criteria. Of these 215 articles, 147 featured cardiovascular conditions among emerging adult populations with a COVID-19 context: 36 and 111 articles from the initial and updated search, respectively. The grey literature search located no additional sources. Altogether, the evidence in this review includes 117 case reports/series,^22^, ^23^, ^24^, ^25^, ^26^, ^27^, ^28^, ^29^, ^30^, ^31^, ^32^, ^33^, ^34^, ^35^, ^36^, ^37^, ^38^, ^39^, ^40^, ^41^, ^42^, ^43^, ^44^, ^45^, ^46^, ^47^, ^48^, ^49^, ^50^, ^51^, ^52^, ^53^, ^54^, ^55^, ^56^, ^57^, ^58^, ^59^, ^60^, ^61^, ^62^, ^63^, ^64^, ^65^, ^66^, ^67^, ^68^, ^69^, ^70^, ^71^, ^72^, ^73^, ^74^, ^75^, ^76^, ^77^, ^78^, ^79^, ^80^, ^81^, ^82^, ^83^, ^84^, ^85^, ^86^, ^87^, ^88^, ^89^, ^90^, ^91^, ^92^, ^93^, ^94^, ^95^, ^96^, ^97^, ^98^, ^99^, ^100^, ^101^, ^102^, ^103^, ^104^, ^105^, ^106^, ^107^, ^108^, ^109^, ^110^, ^111^, ^112^, ^113^, ^114^, ^115^, ^116^, ^117^, ^118^, ^119^, ^120^, ^121^, ^122^, ^123^, ^124^, ^125^, ^126^, ^127^, ^128^, ^129^, ^130^, ^131^, ^132^, ^133^, ^134^, ^135^, ^136^, ^137^, ^138^ 28 observational studies,^10^^,^^11^^,^^139^, ^140^, ^141^, ^142^, ^143^, ^144^, ^145^, ^146^, ^147^, ^148^, ^149^, ^150^, ^151^, ^152^, ^153^, ^154^, ^155^, ^156^, ^157^, ^158^, ^159^, ^160^, ^161^, ^162^, ^163^, ^164^ and 2 reviews/editorials.^165^^,^^166^ Nearly half (49%; n = 72) are from the United States (US), followed by Iran (6.1%; n = 9), the United Kingdom (5.4%; n = 8), and France (5.4%; n = 8).

## Objective #1: Impact of the COVID-19 Pandemic on the Cardiovascular Health of Emerging Adults

Of the 147 articles included, 117 case reports/series identified 123 cases of emerging adults aged 18-25 (mean age = 21; 66% male) with active or previous COVID-19 and cardiovascular presentations/complications, 47 of which were classified by authors as hyperinflammatory syndromes, namely, MIS. Tachycardia (n = 65) was the most common cardiovascular presentation (accompanied by MIS or another cardiovascular complication in 89% of cases), followed by ventricular dysfunction (n = 44), hypotension (n = 37), thrombosis (n = 23), including pulmonary embolism and stroke, cardiomyopathy (n = 28), including myocarditis and myopericarditis, heart failure (n = 27), hypertension (n = 8), bradycardia (n = 9), myocardial infarction (n = 5), and cardiac arrest (n = 4); a detailed report of these cases can be found in Supplemental Table S2. The majority of these cases occurred in emerging adults with no reported history of CVD (91%) or other comorbidities (60%), suggesting that COVID-19 can lead to severe and sometimes fatal cardiovascular health outcomes (8.9%; n = 11) in otherwise healthy young adults. The following describes the types of cardiovascular manifestations in emerging adults who contracted COVID-19 as well as related impacts on cardiovascular care.

### Reports of myocarditis and cardiac abnormalities

In the initial search, 2 observational studies^10^^,^^11^ focusing on COVID-19–associated myocarditis and cardiac abnormalities (ie, findings not meeting the 2018 Lake Louise Criteria^167^) among emerging adults were identified. Starekova et al.^11^ examined electronic health records of 145 university student-athletes aged 17-23 recovering from COVID-19 and found that 1.4% (n = 2) had cardiac magnetic resonance imaging (cMRI) findings consistent with myocarditis. In a cross-sectional study by Brito et al.,^10^ sequential cMRI was performed on 48 college student-athletes (mean age = 19) who experienced mild or asymptomatic COVID-19; cardiac abnormalities were observed in 56% (n = 27) of participants, including pericardial (n = 13), myocardial (n = 8), and myopericardial involvement (n = 6). These results initially suggested that myocarditis and general cardiac effects may be relatively common among healthy emerging adults post-infection. Since then, 10 additional articles have been identified,^139^, ^140^, ^141^, ^142^, ^143^, ^144^, ^145^^,^^147^^,^^165^^,^^166^ providing further insight into the prevalence and associations of myocarditis and cardiac abnormalities after COVID-19 in emerging adults. The majority of these data come from observational studies on student-athlete populations.^139^, ^140^, ^141^, ^142^, ^143^, ^144^, ^145^^,^^165^^,^^166^ In the broader emerging adult population, hospital-based administrative data from 2019 through 2021 from over 900 sites in the US identified 121 inpatients aged 16-24 diagnosed with COVID-19 and myocarditis, yielding an adjusted risk 7.4 times greater than that of their uninfected counterparts (0.098% vs 0.013%, respectively).^147^

A recent systematic review by van Hattum et al.^166^ provided prevalence estimates of COVID-19–associated myocarditis and other cardiac abnormalities (eg, arrhythmias) among student-athletes after infection. Among 2326 college athletes (median age = 22), the weighted prevalence of myocarditis found with cMRI was 2.1% using the established Lake Louise Criteria. The majority (59%) of these individuals were mildly symptomatic while infected; 22% were asymptomatic, and the remainder experienced moderate (19%) or severe (0.2%) illness. There were no observed arrhythmias and only 1 resuscitated cardiac arrest unlikely attributable to COVID-19. Several studies in our evidence set specific to COVID-19–related myocarditis were included in the review;^166^ prevalence estimates ranged from 0% to 15%,^10^^,^^11^^,^^139^, ^140^, ^141^, ^142^, ^143^, ^144^, ^145^, ^146^^,^^165^^,^^166^ which included both asymptomatic and symptomatic cases of COVID-19.

Additional evidence has accumulated regarding the prevalence of general cardiac involvement (eg, myocardial oedema and pericardial effusion) in emerging adults with COVID-19. In a large cohort of 3018 college athletes (mean age = 21 years), 0.7% (95% confidence interval [CI]: 0.4, 1.1) were determined to have definite, probable, or possible severe acute respiratory syndrome coronavirus 2 (SARS-CoV-2)-related myocardial or pericardial involvement with cMRI or “triad” testing: cardiac troponin levels, an electrocardiogram (ECG), and a transthoracic echocardiogram.^140^ An analysis by Petek et al.^141^ used data from the Outcomes Registry for Cardiac Conditions in Athletes (ORCCA), representing 44 US colleges, with a sample of 3597 student-athletes (mean age = 20) diagnosed with COVID-19. Investigation of individuals with exertional cardiopulmonary symptoms (n = 137) revealed 10 cases of cardiovascular sequelae (7.3%) and 5 cases (3.6%) of definite or probable cardiac involvement via cMRI, representing 21% of athletes with chest pain after COVID-19. In another group of 170 athletes aged 18-25, 3.5% (n = 6) had abnormal cardiac rhythms and 1.2% (n = 2) were diagnosed with viral pericarditis using cMRI.^139^ In a sample of 137 student-athletes aged 18-27, 82% (n = 112) of whom were symptomatic, algorithm-guided screening after COVID-19 identified trace pericardial effusions with echocardiography in 2.9% (n = 4) of participants.^143^ Similarly, although Małek et al.^144^ identified no cases of myocarditis via cMRI in a cohort of 26 athletes recovering mainly from asymptomatic or mild infections (median age = 24), 19% (n = 5) demonstrated cardiac abnormalities, including signs of myocardial oedema and pericardial effusion. Compared with myocarditis, most studies suggest that general cardiac involvement among emerging adults following COVID-19 is more common (0%-58%).^10^^,^^11^^,^^139^, ^140^, ^141^, ^142^, ^143^, ^144^, ^145^, ^146^^,^^166^ However, it is of note that data from larger cohorts (ie, N > 100) provided smaller prevalence estimates in athletes imaged with cMRI (0%-7%).^11^^,^^139^, ^140^, ^141^^,^^143^

#### Risk factors

Cohort analyses using cMRI or triad testing identified several risk factors for cardiac involvement, including White Hispanic race (odds ratio [OR]: 7.6; 95% CI: 2.2, 26.1), cardiopulmonary symptoms before or during infection or return to exercise (adjusted OR [aOR]: 3.1; 95% CI: 1.2-7.8), or 1 or more abnormal triad test results potentially associated with COVID-19 (aOR: 37.4; 95% CI: 13.3-105.3).^140^^,^^141^ The association between biological sex and COVID-19–associated cardiac abnormalities (eg, abnormal ECG findings and diagnosis of myocarditis) was unclear.^139^^,^^147^ With respect to age, the risk of COVID-19–associated myocarditis in hospitalized patients was lowest among individuals aged 16-24 when compared with older age cohorts.^147^

Ultimately, our review of the evidence suggests the prevalence of COVID-19–associated myocarditis among otherwise healthy emerging adult student-athletes to be nearing the lower end of 0.5%-3%,^11^^,^^139^, ^140^, ^141^^,^^143^^,^^165^^,^^166^ though general cardiac involvement (eg, myocardial oedema and pericardial effusions) is likely more prevalent (0%-7%),^11^^,^^139^, ^140^, ^141^^,^^143^ particularly in those with lingering cardiopulmonary symptoms.^141^ Other cardiac events, such as arrhythmias and cardiac arrest, seem to be less common in this age cohort after infection.^166^ There was a relatively low risk of clinical cardiac events (ie, significant arrhythmias, heart failure, sudden cardiac arrest, or death) in short-term follow-up (approximately 0.03%).^140^

### Reports of hyperinflammatory syndromes

Belay et al.^148^ conducted the largest US cohort study to date (n = 1733) describing the clinical characteristics and geographical and temporal distribution of patients under 21 years of age with MIS-C. Of the 55 emerging adults aged 18-20, 58% (n = 32) were admitted to the ICU, and 11% (n = 6) died. Cardiovascular manifestations in this age group included hypotension in 53%, cardiac dysfunction in 42%, myocarditis in 31%, pericardial effusion in 27%, and coronary artery dilation or aneurysm in 15%. Compared with the paediatric population, patients aged 18-20 had the highest proportion of myocarditis (31% in 18- to 20-year-olds vs 9.2%-28% in 0- to 17-year-olds; *P* < 0.001). The 18- to 20-year-old cohort, however, had the lowest incidence of MIS at 0.4 per 100,000 infected (*P* < 0.001).

In this review, 47 cases (mean age = 21; 70% male) of emerging adults with cardiovascular complications in the context of confirmed or suspected hyperinflammatory syndromes and active or previous COVID-19 were identified.^22^, ^23^, ^24^, ^25^, ^26^, ^27^, ^28^, ^29^, ^30^, ^31^, ^32^, ^33^, ^34^, ^35^, ^36^, ^37^, ^38^, ^39^, ^40^, ^41^, ^42^, ^43^, ^44^, ^45^, ^46^, ^47^, ^48^, ^49^, ^50^, ^51^, ^52^, ^53^, ^54^, ^55^, ^56^ If not concomitant COVID-19, previous infections typically occurred 3-8 weeks before the onset of symptoms.^23^^,^^30^^,^^32^, ^33^, ^34^^,^^40^^,^^43^, ^44^, ^45^, ^46^^,^^52^^,^^53^^,^^56^ Alongside MIS-C/A, cardiovascular presentations/complications included ventricular dysfunction (n = 33), tachycardia (n = 28), hypotension (n = 16), cardiogenic shock (n = 14), cardiomyopathy (n = 11), including myocarditis and pericarditis, valve insufficiency (n = 4), atrial fibrillation (n = 2), and non–ST-elevation myocardial infarction (n = 2); refer to Supplemental Table S2 for additional case details. Cardiac involvement with echocardiographic changes (eg, reduced ejection fraction and global hypokinesia) and elevated N-terminal pro-B-type natriuretic peptide and troponin were frequently reported among these cases. Of note, 79% (n = 37) of cases were previously healthy with no past medical history, and 98% (n = 46) had no reported history of CVD.

Due to prompt identification and treatment, there were few reports (4.3%; n = 2) of emerging adults with MIS who died.^48^^,^^55^ A case series described the postmortem examinations of 4 deaths due to maternal or paediatric antemortem COVID-19. In this report, the death of a 19-year-old woman was attributed to MIS causing coagulopathy.^55^ A separate retrospective cohort study by Whitworth et al.^149^ identified the incidence of thrombosis in children and adolescents under 21 years of age hospitalized with COVID-19 or MIS-C (n = 564). Eleven of the 20 cases of thrombosis (55%) occurred in patients aged 16-21, 36% (n = 4) of whom died; all cases were either African American or Hispanic individuals. Patients 12 and older with MIS-C displayed the highest rate of thrombotic events (19%). Despite the incidence of MIS among emerging adults being lower than younger age cohorts, those affected seem to be at an elevated risk of cardiovascular complications, including myocarditis, and death. Evidence outlining risk factors and long-term prognosis after recovery in this age group is lacking.

### Reports of vascular alterations

Multiple studies reported signs of vascular dysfunction among young adults after COVID-19.^122^^,^^160^, ^161^, ^162^, ^163^, ^164^

Ratchford et al.^160^ performed a cross-sectional analysis on healthy young adults (mean age = 20) to examine the effects of COVID-19 on markers of vascular function and arterial stiffness. When compared with uninfected individuals, those with COVID-19 experienced significantly lower brachial artery flow-mediated dilation (FMD) (2.7% ± 1.2% vs 8.8% ± 3.0%; *P* < 0.01) and femoral artery blood flow response (−3 ± 91 mL vs 118 ± 114 mL; *P* < 0.01) 3-4 weeks after infection. This same group performed another cross-sectional study^161^ and found higher carotid artery stiffness among young adults with COVID-19 (6 ± 1 m/s) compared with healthy controls (5 ± 1 m/s; *P* = 0.02). Aortic augmentation index was also greater in the COVID-19 group (12.7% ± 9.1% vs 3.3% ± 12.6%; *P* = 0.03), suggesting aortic stiffening and potential atherosclerotic risk progression. Another study by Stute et al.^163^ similarly found that resting muscle sympathetic nerve activity, a measure of arterial stiffness, was higher in individuals recovering from COVID-19 (n = 16; mean age = 20) compared with uninfected controls (285 ± 101 a.u./min vs 159 ± 46 a.u./min, respectively; *P* = 0.001).

Growing evidence indicates that endothelial inflammation associated with COVID-19 may contribute to cerebrovascular disease. A case series by Arandela et al.^122^ identified 2 patients between the ages of 18 and 25 who developed reversible cerebral vasoconstriction syndrome in the context of COVID-19, suggesting a potential risk in this age cohort among those using vasoactive agents (eg, marijuana). Alterations in cerebral and peripheral vasculature were further investigated in a cohort study conducted by Nandadeva et al.^162^ Analysis found that only peripheral vascular function was impaired in young adults with lingering COVID-19 symptoms (n = 8; mean age = 24); this impairment was not seen in those who were no longer symptomatic (n = 8; mean age = 22). Taken together, these findings suggest that the effects of COVID-19 on central large arteries may be a transient phenomenon.

Whereas the previously mentioned studies reported vascular alterations at rest, Stute et al.^164^ aimed to elucidate the effect of COVID-19 on central and peripheral haemodynamics during a rhythmic handgrip exercise. Brachial artery blood flow was significantly lower in the COVID-19 group (n = 13; mean age = 21) compared with controls (n = 13; mean age = 27): 386.3 ± 132.5 mL/min vs 507.4 ± 109.9 mL/min, respectively (*P* = 0.002). The COVID-19 group also displayed greater increases in systolic blood pressure, systolic arterial pressure, and rate pressure product on exertion.

### Reports of individuals with cardiovascular-related vulnerabilities that increase risk for severe COVID-19, related cardiovascular complications, and mortality

#### Cardiovascular disease, hypertension, and obesity

A few studies offered insight into cardiovascular-related risk factors that may confer an increased risk of severe COVID-19. Fathi et al.^157^ developed a model to predict 2-week mortality using data from 57,705 inpatients with COVID-19, which included a “young” cohort (n = 1049; aged 15-24). Among those who died in this subsample (n = 50; 4.8%), hypertension was significantly associated with 2-week mortality (OR: 54.3; 95% CI: 19.9, 168.2). A retrospective study of young adult COVID-19 patients aged 18-35 admitted to New York City public hospitals also found that cardiac comorbidities and hypertension were associated with increased mortality; however, those with these comorbidities in the 18- to 23-year-old cohort all recovered.^155^ To provide context, data from the National Health Interview Survey and an undergraduate student sample during the pandemic show that pre-existing heart conditions affect 0.5%-1.9% of this age group.^153^^,^^156^ Furthermore, evidence from after a COVID-19 lockdown found that the prevalence of hypertension among undergraduate students (n = 325; mean age = 22) remained at 1%.^154^

A hospital system-based retrospective chart review^159^ conducted in Texas identified risk factors for severe disease and readmission among young adults aged 18-29 diagnosed with COVID-19 (mean age = 24). The study identified 1853 patients with COVID-19, 8% (n = 148) of whom experienced a composite disease outcome (eg, a severe respiratory or cardiovascular event) within 30 days of their first encounter. In this cohort of young adults, older age, obesity, previous CVD (ie, myocardial infarction, congestive heart failure, and cerebrovascular disease), and diabetes were among significant risk factors for composite disease outcomes (*P* ≤ 0.03). A history of CVD and obesity were also predictors of severe disease and/or readmission within 30 days (*P* < 0.05). In addition, the authors highlighted the relationship between race and ethnicity (eg, Hispanic ethnicity) and poorer health outcomes. A preliminary analysis (abstract) by Sands-Lincoln et al.^168^ of patients aged 18-24 with COVID-19 (n = 6648; mean age = 22) found African American (OR: 2.4; 95% CI: 1.6, 3.5) and “other race” identity (OR: 5.0; 95% CI: 2.6, 9.1), CVD (OR: 4.0; 95% CI: 2.8, 5.7), and obesity (OR: 3.0; 95% CI: 2.1, 4.3) to be associated with increased odds of hospitalization. Another study by Richardson et al.^158^ analysed data from hospitalized patients aged 18-39 at acute care hospitals in New York City. Notably, among patients aged 18-24 (n = 119), those who died (n = 4) or required invasive mechanical ventilation (n = 7) were all obese, and 5 of these patients had additional comorbidities, including Down syndrome and congestive heart failure.

In summary, the majority of cardiovascular-related medical vulnerabilities in emerging adults are rare;^153^^,^^154^^,^^156^ however, when present, data suggest that the risk for severe COVID-19, related cardiovascular complications, and mortality in this age group is not insignificant, particularly among those with comorbidities.

#### Congenital heart disease and genetic syndromes

Predicting the COVID-19 response in emerging adults with existing congenital heart disease (CHD) is challenging given the heterogeneity of the population. Two separate retrospective reviews investigating factors associated with severe COVID-19 and mortality across the CHD population found that the presence of a structural congenital heart defect did not confer an increased morbidity or mortality risk.^150^^,^^151^ Lewis et al.^150^ detailed the experience of 4 emerging adults aged 21-25 with CHD who were described to have moderate-to-severe COVID-19. These individuals did not appear to be disproportionately impacted unless they were at an advanced physiological stage (ie, class C or D) (OR: 19.4) and/or had genetic syndromes (OR: 35.8), such as Down syndrome and DiGeorge syndrome (*P* ≤ 0.002). Similarly, Broberg et al.^151^ found that a worse physiological stage of CHD (eg, Eisenmenger physiology and cyanosis) was associated with mortality (*P* = 0.001), whereas anatomic complexity or defect group was not. These findings suggest that susceptibility to severe COVID-19 among emerging adults with CHD is based primarily on physiological factors, and, when accompanied by certain genetic conditions, such as Down syndrome and DiGeorge syndrome, CHD is associated with increased hospitalization from COVID-19.^71^^,^^150^, ^151^, ^152^^,^^158^

The role of genetic disorders in COVID-19 severity among emerging adults with CVD seems to vary depending on the underlying disorder. Adults with Duchenne muscular dystrophy (DMD) are at risk for cardiorespiratory compromise (ie, cardiomyopathy) and so were thought to be vulnerable to worse COVID-19 outcomes.^138^ However, Quinlivan et al.^138^ reported on 5 emerging adult males aged 18-23 with DMD who contracted COVID-19 and did not develop moderate or severe disease. Despite a history of moderate-to-severe cardiomyopathy, long-term immunosuppressive treatment, and respiratory insufficiency, all patients recovered fully with no complications. This evidence indicates that emerging adults with DMD may not be at an elevated risk of severe COVID-19 and related cardiovascular complications.

### Impacts on cardiovascular care

Cardiovascular care for emerging adults was impacted by the COVID-19 pandemic, which in some cases affected cardiovascular outcomes. For example, Warraich et al.^130^ described a 19-year-old patient who delayed treatment by self-isolating for 2 weeks during the pandemic due to a persistent cough that was later understood to be a symptom of a posterior circulation ischemic (POCI) stroke. Other reports of emerging adults with cardiovascular presentations, including stroke,^130^^,^^132^ intracardiac thrombosis,^135^ pulmonary hypertension,^131^ and sinus tachycardia with atrioventricular block,^133^ found that many avoided seeking treatment due to lockdown measures or fear of contracting COVID-19. In addition, the pandemic resulted in misguided clinical judgement and management. In the case presented by Warraich et al.,^130^ the young patient with POCI stroke lacked risk factors for stroke, and so an initial diagnosis of COVID-19 was made. In another case, a 19-year-old man presenting to the emergency department with constitutional symptoms was repeatedly tested for COVID-19, treated with antibiotics for COVID-19 pneumonia, discharged, and later diagnosed with Coxsackie A myocarditis on readmission.^134^ Similarly, Balfe et al.^137^ presented the case of an 18-year-old girl suffering from rheumatic mitral stenosis whose condition was worsened by the intravenous fluids she was administered under the presumption that she had COVID-19. Reports were also identified in which life-saving cardiovascular care for emerging adults (eg, extracorporeal cardiopulmonary resuscitation) was nearly prevented or complicated due to the patient’s COVID-19 status.^60^^,^^63^^,^^136^ Overall, these cases highlight evidence of crucial cardiovascular care being delayed, misguided, or complicated as a result of the COVID-19 pandemic influencing public and clinical decision-making.

## Objective #2: Strategies to Screen for and Manage Cardiovascular Complications in Emerging Adults

### Myocarditis and cardiac abnormalities

The majority of evidence surrounding screening protocols for COVID-associated myocarditis and cardiac abnormalities in emerging adults focuses on student-athletes.^10^^,^^11^^,^^139^, ^140^, ^141^, ^142^, ^143^, ^144^, ^145^, ^146^^,^^165^^,^^166^

Initial evidence explored the utility of extensive cardiorespiratory and haematological screening in athletes recovering from COVID-19 to identify postinfection cardiovascular abnormalities. Gervasi et al.^146^ examined a cohort of 30 professional soccer players aged 19-27, 18 of whom tested positive for SARS-CoV-2 IgG antibodies and reported previous asymptomatic or mild COVID-19. After comprehensive screening (eg, blood tests, spirometry, and ECG), none of the participants demonstrated clinically relevant cardiovascular abnormalities (eg, myocarditis). Furthermore, both Starekova et al.^11^ and Brito et al.^10^ conducted studies in which university athletes recovering from COVID-19 underwent screening with cMRI, most of whom were recovering from mild-to-moderate or asymptomatic illness. Starekova et al.,^11^ with a sample of athletes aged 17-23, found that only 1.4% (n = 4) of those screened met the Lake Louise Criteria for myocarditis. Brito et al.^10^ identified myocardial, pericardial, or myopericardial abnormalities in 58% of athletes, but no signs of ongoing myocarditis. Because of the low prevalence of clinical myocarditis in these cohorts, cMRI as a standard screening tool for myocarditis was deemed unwarranted, especially for individuals with asymptomatic or mild COVID-19 and those with a normal ECG and cardiac troponin.

Evidence from the updated search continues to support cMRI as a sensitive and specific screening modality for myocarditis and other cardiac abnormalities in this population.^140^, ^141^, ^142^^,^^145^^,^^165^^,^^166^ Still, larger cohort studies recommend cMRI only for individuals with a heightened risk based on an initial, comprehensive cardiac evaluation.^139^, ^140^, ^141^, ^142^, ^143^, ^144^ Multiple studies screened for elevated cardiac troponin^139^, ^140^, ^141^, ^142^, ^143^, ^144^^,^^165^ or abnormal ECG or echocardiogram findings^139^, ^140^, ^141^^,^^143^^,^^144^^,^^165^ to indicate cMRI for further diagnostic workup. A sample of 1597 athletes screened for post-COVID-19 cardiac abnormalities demonstrated the utility of 4 respective screening strategies:^165^^,^^169^

1. A positive cardiac history (eg, chest pain) to indicate triad testing; abnormal triad testing to indicate cMRI
2. Abnormal triad testing to indicate cMRI
3. A positive cardiac history (eg, chest pain) or abnormal triad testing to indicate cMRI
4. cMRI without prior screening.

These approaches would have identified 5 (0.3%), 13 (0.8%), 17 (1.1%), and 37 (2.3%) cases of myocarditis in the cohort, respectively. Moulson et al.^140^ similarly found that 82% of athletes diagnosed with definite or probable cardiac involvement after COVID-19 would have been identified with a stepwise approach using moderate symptom severity, cardiopulmonary symptoms, or abnormal triad testing to indicate cMRI. Despite such evidence showcasing an increased specificity when screening algorithms included a cardiac history and triad testing,^140^^,^^165^^,^^169^ the previously discussed systematic review by van Hattum et al.^166^ found that, among 10 studies (n = 4171), there was no clear association between postrecovery cardiac troponin levels and cardiac abnormalities. In contrast, a comprehensive history appears to have clinical utility, as the Moulson et al.^140^ and Petek et al.^141^ analyses of data from more than 40 US academic institutions found cardiopulmonary symptoms, specifically chest pain and dyspnea, to increase the risk of COVID-19–related cardiac involvement (aOR: 3.1; 95% CI: 1.2-7.8). Of those who had chest pain after return to exercise, 21% had evidence of myocardial and/or pericardial involvement on cMRI.^141^ Ultimately, the impact of these screening algorithms on the clinical course of emerging adult athletes recovering from COVID-19 remains unknown, as few, if any, adverse cardiac events have been reported among this population following return to play.^140^^,^^165^^,^^166^

### MIS-C/A

Regarding the identification of MIS-C/A in emerging adults with concomitant or previous COVID-19, Belay et al.^148^ found that only 71% of the 18- to 20-year-old MIS cases demonstrated SARS-CoV-2 polymerase chain reaction (PCR) positivity, and 58% had positive serology. In our review of MIS case reports/series involving cardiovascular complications, 47% (n = 17) reported negative PCR results, whereas 70% (n = 33) had positive serology. Given the significant proportion of patients with MIS and negative PCR results, the authors have recommended SARS-CoV-2 antibody testing to classify MIS patients without active COVID-19 or those with atypical COVID-19 presentations.^24^ Compared with younger age groups, 18- to 20-year-olds were more likely to report COVID-19–like illness 7 or more days before MIS onset (63% vs 18%-44%; *P* < 0.001).^148^ In the cohort study by Belay et al.,^148^ mainstay treatments for MIS included intravenous immunoglobulin (IVIG) and steroids. Across the 47 cases included in this review,^22^, ^23^, ^24^, ^25^, ^26^, ^27^, ^28^, ^29^, ^30^, ^31^, ^32^, ^33^, ^34^, ^35^, ^36^, ^37^, ^38^, ^39^, ^40^, ^41^, ^42^, ^43^, ^44^, ^45^, ^46^, ^47^, ^48^, ^49^, ^50^, ^51^, ^52^, ^53^, ^54^, ^55^, ^56^ 70% (n = 33) were treated with steroids (eg, methylprednisolone, prednisolone, and prednisone) and 62% (n = 29) with IVIG. Additional therapies included interleukin-1 and interleukin-6 receptor antagonists and supportive care measures, such as aspirin and anticoagulation (Supplemental Table S2).

## Discussion

**Figure 2:**
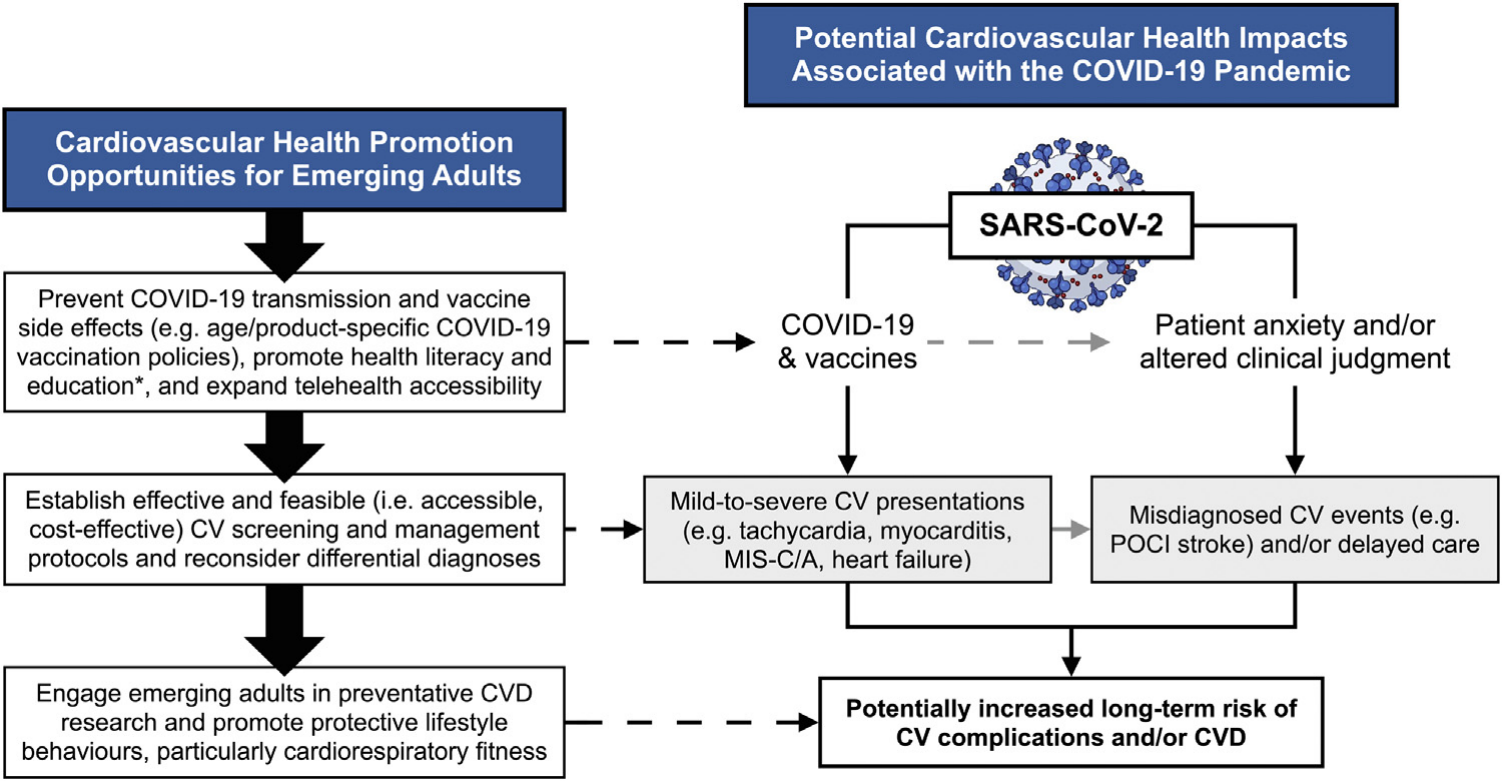
A framework to promote cardiovascular health among emerging adults in the context of the COVID-19 pandemic (created in part with BioRender.com). The **dashed lines** represent pathways to prevent or manage COVID-19–related cardiovascular health concerns. ∗Health literacy and education initiatives may benefit from including parents/guardians as part of the target audience as they often play a role in an emerging adult’s decision to seek medical care. CV, cardiovascular; CVD, cardiovascular disease; MIS-C/A, multisystem inflammatory syndrome in children/adults; POCI, posterior circulation ischemic; SARS-CoV-2, severe acute respiratory syndrome coronavirus 2.

This scoping review examined current literature to describe the impact of the COVID-19 pandemic on the cardiovascular health and care of emerging adults. We identified multiple cases of cardiovascular presentations (eg, tachycardia and ventricular dysfunction), cardiac abnormalities (eg, myocardial/pericardial involvement), MIS-C/A, and vascular alterations among emerging adults who contracted COVID-19, the long-term effects of which remain unknown. The evidence is insufficient to determine the true incidence and prevalence of these complications in this age cohort; however, prevalence estimates from student-athlete samples of COVID-19–associated myocarditis and cardiac involvement on cMRI ranged from 0.5% to 3%^11^^,^^139^, ^140^, ^141^^,^^143^^,^^165^^,^^166^ and 0% to 7%,^11^^,^^139^, ^140^, ^141^^,^^143^ respectively. In some groups, medical vulnerabilities such as obesity,^158^^,^^159^^,^^168^ hypertension,^155^^,^^157^ previous CVD,^159^^,^^168^ and certain genetic syndromes^71^^,^^150^^,^^152^^,^^158^ posed an increased risk of severe illness, cardiovascular complications, and/or mortality from COVID-19. There were also reports of emerging adults with cardiovascular presentations where the provision of cardiovascular care was negatively impacted due to COVID-19.^60^^,^^130^, ^131^, ^132^, ^133^, ^134^, ^135^^,^^137^ Regarding the appropriate screening and management of these cardiovascular abnormalities, the majority of age-specific evidence pertains to COVID-19–associated myocarditis and MIS. Based on the findings of this scoping review, a framework that highlights the negative impacts of the COVID-19 pandemic on the cardiovascular health of emerging adults and corresponding health promotion opportunities to prevent and mitigate these effects was constructed (see Fig. 2).

In the following, accumulated evidence from the review is highlighted with corresponding suggestions for future research, policy, and practice.

### Investigations surrounding COVID-19 and cardiovascular health

In this review, cardiovascular presentations among emerging adults with current or previous COVID-19 ranged from milder manifestations such as sinus tachycardia,^170^ to more serious complications, including myocarditis, stroke, cardiogenic shock, heart failure, thrombosis, and MIS. Investigators suggest that these cardiovascular complications may lead to cardiomyopathy, cardiac arrhythmias, and sudden cardiac arrest in the long term.^169^^,^^171^, ^172^, ^173^ Therefore, ongoing research involving emerging adults who contract COVID-19 and develop cardiovascular sequelae is recommended. Long-term surveillance of these patients can help assess and inform cardiovascular screening and treatment protocols. An emphasis should also be placed on ensuring that study samples are representative of the broader emerging adult population, given most of the cohort evidence found in this review focused on student-athletes.^10^^,^^11^^,^^139^, ^140^, ^141^, ^142^, ^143^, ^144^, ^145^^,^^148^^,^^165^^,^^166^

#### Myocarditis and cardiac abnormalities

Extrapolating prevalence estimates of COVID-19–associated myocarditis and cardiac abnormalities from student-athlete cohorts to the entire emerging adult population is cautioned. Experts believe that athletes who exercise with COVID-19 are at an increased risk of developing myocarditis.^169^^,^^174^ At the same time, their overall heightened cardiovascular health is not representative of the broader population. Regarding the long-term implications of these findings, updated ORCCA data^175^ demonstrate complete or partial resolution of cMRI abnormalities in 80% (n = 8) of participants. Follow-up after more than 1 year identified no adverse cardiac outcomes in the subsample of athletes with initial cardiac involvement. Though promising, additional surveillance is required to establish the true long-term risk of COVID-19–associated myocarditis and other cardiovascular sequelae in emerging adults.

With respect to screening, data from student-athletes may be used to aid clinical decision-making given limited evidence from the broader emerging adult population. Additional analysis of the ORCCA cohort^176^ continues to indicate the limited diagnostic utility of cardiac troponin as a screening modality for those with COVID-19–associated myocardial involvement; it is recommended only for those with a high clinical pretest probability of disease. Moreover, the data in this review do not support routine cMRI in lower-risk individuals.^10^^,^^11^^,^^139^, ^140^, ^141^, ^142^, ^143^, ^144^^,^^146^ This approach would be quite costly and increase the likelihood of false positives.^165^^,^^177^ Rather, a screening approach guided by cardiac symptoms (eg, chest pain) and/or triad testing to indicate cMRI will increase its diagnostic yield and feasibility.^140^^,^^169^ A 2022 expert consensus paper from the American College of Cardiology^178^ is in agreement, recommending against cardiac testing for asymptomatic cases, those with only mild-to-moderate noncardiopulmonary symptoms, or those with previous COVID-19 in the absence of ongoing cardiopulmonary symptoms. Individuals with COVID-19–related cardiopulmonary symptoms are recommended for triad testing and, if results are abnormal, a cardiology consultation to consider subsequent cMRI and additional cardiac testing for diagnosis. The body of evidence in this review is limited with respect to treating COVID-19–associated myocarditis in this age group. However, case reports^66^^,^^75^^,^^98^^,^^120^ and supporting literature^179^ indicate that in the absence of additional cardiovascular complications (eg, acute heart failure), rest, supportive measures (eg, intravenous/oral hydration, beta-blockers), and immunosuppressive therapy yield promising outcomes.

#### MIS-C/A

Emerging adults with MIS demonstrated significant mortality (Supplemental Table S2) and were more often reported to have COVID-19–like illness before presentation and subsequent findings of myocarditis.^148^ Ultimately, these findings emphasize the importance of recognizing and including MIS as a differential in the context of recent COVID-19 in this cohort.

As of now, it remains unclear whether MIS is a manifestation of acute COVID-19 or an entirely postacute phenomenon. Given that not all MIS patients present with positive PCR test results, SARS-CoV-2 antibody testing is recommended as a potentially crucial diagnostic measure to classify and recognize these patients.^24^ However, in the current state of the pandemic, the majority of adults demonstrate seroprevalence of SARS-CoV-2 antibodies,^180^ limiting its clinical utility. The additional use of laboratory tests for inflammation, hypercoagulability, and organ damage (eg, C-reactive protein, D-dimer, and cardiac enzymes) may assist in early identification and subsequent management. To our knowledge, no consensus guidelines are available for MIS-A; current recommendations are extrapolated from evidence specific to MIS-C, applicable to ages less than 21 years. The Centers for Disease Control and Prevention (CDC) supports serologic testing in addition to PCR testing when feasible, as well as workup for cardiac involvement in affected individuals.^181^ Both the CDC and National Institutes of Health discuss IVIG in combination with glucocorticoids (eg, methylprednisolone) as the first-line treatment strategy, citing its benefits for faster recovery of cardiac function.^181^^,^^182^ The latest clinical guideline from the American College of Rheumatology additionally recommends higher doses of steroids, anakinra, or infliximab for refractory disease.^183^ Future research into MIS is required to better elucidate pertinent risk factors, facilitate diagnosis and management, and identify any long-term cardiovascular implications in this age group.

#### Changes in systemic vasculature

Reports of young adults recovering from COVID-19 found transient alterations in arterial, cerebral, and peripheral vasculature in those with lingering symptoms and even after recovery.^122^^,^^160^, ^161^, ^162^, ^163^, ^164^ Participants in these studies were all assessed 3-8 weeks after their first positive COVID-19 test; therefore, there is limited long-term data to substantiate these findings. Of note, 1 study reported a 6% difference in brachial artery FMD among those with previous COVID-19 compared with those who were uninfected.^160^ A meta-analysis by Inaba et al.^184^ (mean age > 50) found that for every 1% decrease in brachial artery FMD, there is a corresponding 8% increase in risk of future cardiovascular events, including stroke, heart attack, and death. In addition, these findings represent dysautonomia after COVID-19, a phenomenon observed among those experiencing “long COVID,” the symptoms of which also include fatigue and shortness of breath.^185^, ^186^, ^187^ These sequelae may lead to exercise intolerance among those recovering from COVID-19 and pose future implications for CVD risk among emerging adults. Moving forward, follow-up in this group is needed to better understand the relationship between COVID-19 and long-term vascular function.

#### Risk stratification of emerging adults for COVID-19 severity, related cardiovascular complications, and mortality

There is increasing evidence that certain cardiovascular comorbidities (eg, obesity, hypertension, CVD)^153^, ^154^, ^155^, ^156^, ^157^, ^158^, ^159^^,^^168^ are relevant risk factors among emerging adults in the context of COVID-19. Among emerging adults with CHD who contract COVID-19, these patients may still be considered high-risk due to the variability in this population’s clinical presentation and response to treatments.^150^^,^^151^ In the future, retrospective analyses should focus on emerging adults who have experienced severe COVID-19 and related cardiovascular complications to further clarify the relationship between these outcomes and presumptive risk factors, particularly CVD. Because most sample sizes in the literature were modest, it is recommended that multicentre registries specific to emerging adults, such as the ORCCA^141^ and COVID-19 CVD Registry,^188^ be established to enable the aggregation of data and provide adequate statistical power to conduct such analyses.

### The cardiovascular safety of COVID-19 vaccines for emerging adults and public education

As vaccination efforts progress around the globe, there have been signals of adverse cardiovascular events from COVID-19 vaccines, specifically cases of myocarditis and pericarditis among emerging adults, which were not observed in clinical trials.^16^^,^^17^^,^^189^ In a sample of approximately 23 million Nordic residents, the number of excess cases of myocarditis among males aged 16-24 associated with a second dose of the BNT162b2 (Pfizer) and mRNA-1273 (Moderna) vaccines 28 days after administration was estimated at 4-7 and 9-28 per 100,000 doses, respectively.^16^ A US study using the Vaccine Adverse Event Reporting System (VAERS) data^17^ similarly found an elevated risk among males aged 18-24, with 52 and 56 cases of myocarditis per million after second doses of the Pfizer and Moderna vaccine, respectively; comparatively, there were only 4-7 cases of myocarditis per million doses in the female cohort. No reported deaths in vaccinated individuals younger than 30 were attributed to myocarditis, apart from 1 potential case. Of note, VAERS reports can be submitted by any member of the public and are not verified for cause-and-effect relationships, making this passive surveillance system particularly susceptible to false reports and under- or over-reporting. Still, meta-analysis reveals the incidence of myopericarditis associated with COVID-19 vaccines to be either comparable to or lower than that of other vaccines.^189^

The cardiovascular safety concerns of COVID-19 vaccines must be contextualized with the cardiovascular risks associated with COVID-19 infection. A recent CDC analysis found that the risk of cardiovascular complications (ie, myocarditis, pericarditis, or MIS) among males aged 18-29 was 7-8 times greater after COVID-19 compared with vaccination; a similar observation was found for females.^190^ A population-based cohort study^191^ of individuals in Ontario, Canada, found that the highest rate was among males aged 18-24 receiving Moderna vs Pfizer as the second dose (300 vs 59 cases per million doses, respectively) and was significantly higher when the interdose interval was ≤30 days compared with ≥56 days (95-377 vs 11-132 per million doses, respectively). In addition, UK data^192^ found an association between COVID-19 and pericarditis and cardiac arrhythmias among 16- to 29-year-olds, though this same association was not established with vaccines. For additional context, data suggest that 1 million second mRNA COVID-19 vaccine doses could prevent 11,000 cases, 560 hospitalizations, 138 ICU admissions, and 6 deaths among 12- to 29-year-olds, compared with 39-47 potential cases of vaccine-related myocarditis.^193^ Vaccine-related myocarditis also tends to be milder when compared with COVID-19–related cases, which have been associated with a greater risk of hospitalization and death.^192^

Concerns regarding cardiovascular complications following COVID-19 in this cohort are partly due to the fact that emerging adults contract COVID-19 at disproportionately higher rates than older adults.^156^ Though in agreement with the recommendation for COVID-19 vaccination in this cohort, we encourage continued surveillance and analysis of data in this area.^194^ This measure is especially important in light of waning vaccine immunity, increasing natural immunity, and the emergence of new SARS-CoV-2 variants that seem to pose a lower risk of hospitalization and severe disease.^195^ Therefore, a risk-benefit analysis of COVID-19 vaccines should be regularly updated for emerging adults as the pandemic progresses. To mitigate public hesitancy, as well as the cardiovascular risks associated with COVID-19 vaccines and infection in this age group, we recommend researchers, policymakers, and clinicians consider the following:

• The development and distribution of strain-specific boosters that improve vaccine efficacy against transmission and symptomatic illness;^196^^,^^197^
• Longer COVID-19 vaccine interdose intervals and age-based product considerations^191^ to reduce the incidence of cardiovascular side effects;
• Ongoing surveillance of COVID-19 and vaccine-associated cardiovascular sequelae (eg, myocarditis, pericarditis, and MIS);^15^^,^^194^ and
• Clinician and public education (for emerging adults and parents/guardians) surrounding the signs and symptoms and expected timelines of both infection- and vaccine-associated cardiovascular sequelae; see Table 1.

**Table 1:** Signs and symptoms of COVID-19–associated cardiovascular sequelae that affect emerging adults and recommendations for when to seek medical care, as per the Centers for Disease Control and Prevention^15^^,^^194^

Cardiovascular sequelae	Recommendation
Myocarditis and pericarditis	Seek medical care if you or your child have any of the following symptoms after COVID-19 infection or vaccination:∗ • Chest pain • Shortness of breath • Feelings of having a fast-beating, fluttering, or pounding heart
MIS-C/A	Seek medical care if you or your child have any of the following signs/symptoms after COVID-19:† • Ongoing fever PLUS >1 of the following: ○ Stomach pain ○ Bloodshot eyes ○ Diarrhoea ○ Dizziness or lightheadedness ○ Skin rash ○ Vomiting
	Seek emergency medical attention if you or your child have any of the additional signs/symptoms:† • Trouble breathing • Persistent pain or pressure in the chest • New confusion • Inability to wake or stay awake • Pale-, grey-, or blue-coloured skin, lips, or nail beds (depending on skin tone)

MIS-C/A, multisystem inflammatory syndrome in children/adults.

∗ More often after the second dose, usually within 1 week after vaccination (median time = 3 days).^191^

† Usually within 6 weeks after infection or exposure.

### Preventing misdiagnoses, delayed care, and future COVID-19–related CVD

This review suggests that when emerging adults present with COVID-19–like symptoms, the differential diagnosis should include potentially life-threatening cardiovascular manifestations, such as POCI stroke and other forms of viral myocarditis.^130^^,^^134^^,^^137^ In addition, emerging adults who have cardiovascular symptoms, along with their parents/guardians, need to be educated on the critical importance of seeking medical care despite the risk of contracting COVID-19.^130^, ^131^, ^132^, ^133^, ^134^, ^135^^,^^137^ The increasing availability of telehealth and digital health interventions^198^ may assist younger patients in receiving timely diagnoses and treatment for potentially life-threatening cardiovascular events.

From a developmental perspective, emerging adulthood is a critical time to establish behaviours that promote long-term cardiovascular health. In a national longitudinal study by Clark et al.,^199^ the average 30-year risk of developing CVD for young adults (mean age = 29) ranged from 4.4% to 18%. Health literacy and education initiatives^198^^,^^200^^,^^201^ that emphasize CVD prevention (eg, healthy diets, physical activity, and avoiding substance use) and the recognition of cardiovascular events need to be developed and targeted at emerging adults and parents/guardians.^202^, ^203^, ^204^, ^205^ Promoting cardiorespiratory fitness is also an integral aspect of CVD prevention. An analysis of over 1.5 million Swedish men conscripted to the military (mean age = 18.3) found that higher cardiorespiratory fitness in late adolescence and early adulthood was significantly protective against COVID-19–associated hospitalization (OR: 0.76), ICU admission (OR: 0.61), and mortality later in life (OR: 0.56).^206^ Encouraging cardiorespiratory fitness among emerging adults now will likely improve cardiovascular outcomes if faced with future pandemics.

### Strengths and limitations

The strengths of this review include its comprehensive search strategy, an updated search, and an in-depth analysis of the acquired evidence. As per scoping review guidelines,^20^ included articles were not critically appraised. Therefore, any conclusions drawn from this evidence set should act as a foundation, rather than a guideline, for future research, policy, and practice. The authors also acknowledge the potential for bias and subjectivity in how data were reported given the qualitative nature of scoping reviews. In addition to the writing team including multiple members as a means of limiting individual bias, data are reported thoroughly in the supplemental files and available for review. The major limitation of this review, however, is that the majority of available cohort analyses focused on student-athlete populations; this reduces the generalizability of our findings to the broader emerging adult population. In addition, despite including articles from non–English-speaking countries when available in English, excluding non–English-language articles likely skews the evidence set and prevents extrapolation to the global population. Moreover, the COVID-19 pandemic has been dynamic with respect to the transmissibility and pathogenicity of the current variants at large, rendering older evidence less relevant. Nonetheless, this review provides a novel perspective and foundation for future research, policy, and practice.

## Conclusions

To the best of our knowledge, this review is the first to focus on studies that describe the impact of COVID-19 on the cardiovascular health of emerging adults. Among otherwise healthy emerging adults who contracted COVID-19, rare and sometimes fatal cardiovascular presentations were reported. Compared with younger age cohorts, MIS in emerging adults has been associated with a greater risk of cardiovascular involvement, specifically myocarditis, thrombosis, and mortality. Limited data for emerging adults suggest that obesity, hypertension, CVD, and/or belonging to marginalized groups increase the risk of severe COVID-19, related cardiovascular complications, and mortality. Future research needs to further define the prevalence and risk factors of COVID-19–associated cardiovascular complications in this demographic. Screening and treatment protocols for these complications (eg, triad testing and cMRI) still require further development and validation using data from the broader emerging adult population. Within health care practice, several measures are encouraged to foster long-term cardiovascular health among emerging adults: (i) conducting differential diagnoses for cardiovascular issues in those with COVID-19–like symptoms; (ii) promoting health literacy and education among emerging adults and parents/guardians, including cardiorespiratory fitness; and (iii) expanding telehealth accessibility. Although COVID-19 vaccines are still recommended for emerging adults, policymakers and clinicians should be aware of the potential cardiovascular risks and use the most recent surveillance data to guide vaccination policies for this age group. These actions should prove useful while the scientific community continues to unravel the long-term cardiovascular implications of COVID-19 among emerging adults.
