# Supplementary material for: The Impact of COVID-19 on the Cardiovascular Health of Emerging Adults Aged 18-25: Findings From a Scoping Review
Source: CJC Pediatr Congenit Heart Dis. 2022 Dec 1;2(1):33–50. doi: 10.1016/j.cjcpc.2022.11.005 (PMC9711905; doi:10.1016/j.cjcpc.2022.11.005)
Supplement: Supplementary Data [file mmc1.pdf]

Supplemental File

Supplemental Table S1. Search strategies and number of results by bibliographic database.

Note: The following search strategies additionally include key terms for cardiovascular disease risk factors (eg, tobacco use) and mental health topics (eg, depression) as this scoping review was part of a larger undergraduate thesis project.

Database	Search Strategy	Number of Results
MEDLINE (via Ovid)	“(Coronavirus Infections/ or COVID19.mp. or COVID-19.mp. or "coronavirus disease 2019".mp. or SARS-CoV-2.mp. or "severe acute respiratory syndrome coronavirus 2".mp. or 2019-nCoV.mp. or "novel coronavirus".mp.) and (Young Adult/ or Students/ or "emerging adult*".mp. or "young adult*".mp. or student*.mp. or universit*.mp. or college*.mp. or undergraduate*.mp.) and (exp Hypertension/ or exp Arrhythmias, Cardiac/ or exp Heart Failure/ or exp Heart Arrest/ or exp Myocardial Ischemia/ or exp Stroke/ or Ischemic Attack, Transient/ or exp Heart Valve Diseases/ or exp Cardiomyopathies/ or exp Cardiovascular Abnormalities/ or cardiovasc*.mp. or heart.mp. or hypertension.mp. or "high blood pressure*".mp. or arrhythmia*.mp. or myocar*.mp. or coronar*.mp. or ischem*.mp. or stroke*.mp. or cerebrovasc*.mp. or valv*.mp. or cardiomyopath*.mp. or exp Exercise/ or exp Recreation/ or "physical activit*".mp. or exercis*.mp. or recreation*.mp. or sport*.mp. or exp "Tobacco Use"/ or "Tobacco Use Disorder"/ or exp Alcohol-Related Disorders/ or Alcohol Drinking/ or Alcohol Drinking in College/ or tobacco.mp. or alcohol*.mp. or Depression/ or Depressive Disorder/ or Depressive Disorder, Major/ or Dysthymic Disorder/ or depress*.mp. or dysthymi*.mp.)”	n=3069 (1522+1547)
Embase	(sars-related coronavirus/ or COVID19.mp. or COVID-19.mp. or "coronavirus disease 2019".mp. or SARS-CoV-2.mp. or "severe acute respiratory syndrome coronavirus 2".mp. or 2019-nCoV.mp. or "novel coronavirus".mp.) and (young adult/ or student/ or university student/ or college student/ or undergraduate student/ or "emerging adult*".mp. or "young adult*".mp. or student*.mp. or universit*.mp. or college*.mp. or undergraduate*.mp.) and (exp elevated blood pressure/ or exp heart arrhythmia/ or exp ischemic heart disease/ or exp heart failure/ or exp cerebrovascular accident/ or transient ischemic attack/ or exp valvular heart disease/ or exp cardiomyopathy/ or exp myocarditis/ or exp cardiovascular malformation/ or cardiovasc*.mp. or heart.mp. or hypertension.mp. or "high blood pressure*".mp. or arrhythmia*.mp. or myocar*.mp. or coronar*.mp. or ischem*.mp. or stroke*.mp. or cerebrovasc*.mp. or valv*.mp. or cardiomyopath*.mp. or exp physical activity/ or exp exercise/ or exp recreation/ or exp sport/ or "physical activit*".mp. or exercis*.mp. or recreation*.mp. or sport*.mp. or exp "tobacco use"/ or tobacco dependence/ or alcohol consumption/ or drinking behavior/ or exp alcohol abuse/ or exp alcoholism/ or tobacco.mp. or alcohol*.mp. or depression/ or major depression/ or dysthymia/ or depress*.mp. or	n=4590 (1600+2990)

	dysthymi*.mp.)	
Web of Science (Core Collection)	(COVID19 OR COVID-19 OR "coronavirus disease 2019" OR SARS-CoV-2 OR "severe acute respiratory syndrome coronavirus 2" OR 2019-nCoV OR "novel coronavirus") AND ("emerging adult*" OR "young adult*" OR student* OR universit* OR college* OR undergraduate*) AND (cardiovasc* OR heart OR hypertension OR "high blood pressure*" OR arrhythmia* OR myocar* OR coronar* OR ischem* OR stroke* OR cerebrovasc* OR valv* OR cardiomyopath* OR "physical activit*" OR exercis* OR recreation* OR sport* OR tobacco OR alcohol* OR depress* OR dysthymi*)	n=1707 (822+885)
CINAHL	(MH "COVID-19" OR COVID19 OR COVID-19 OR "coronavirus disease 2019" OR SARS-CoV-2 OR "severe acute respiratory syndrome coronavirus 2" OR 2019-nCoV OR "novel coronavirus") AND (MH "Young Adult" OR MH "Students" OR MH "Students, Undergraduate" OR MH "Students, College" OR "emerging adult*" OR "young adult*" or student* OR universit* OR college* OR undergraduate*) AND (MH "Hypertension+" OR MH "Arrhythmia+" OR MH "Myocardial Ischemia+" OR MH "Heart Failure+" OR MH "Heart Arrest+" OR MH "Stroke+" OR MH "Cerebral Ischemia, Transient" OR MH "Heart Valve Diseases+" OR MH "Myocardial Diseases+" OR MH "Cardiovascular Abnormalities+" OR cardiovasc* OR heart OR hypertension OR "high blood pressure*" OR arrhythmia* OR myocar* OR coronar* OR ischem* OR stroke* OR cerebrovasc* OR valv* OR cardiomyopath* OR MH "Physical Activity" OR MH "Exercise+" OR MH "Recreation+" OR MH "Sports+" OR MH "Smoking" OR MH "Alcohol Drinking+" OR MH "Alcohol-Related Disorders+" OR "physical activit*" OR exercis* OR recreation* OR sport* OR tobacco OR alcohol* OR MH "Depression" OR MH "Dysthymic Disorder" OR depress* OR dysthymi*)	n=770 (425+345)
PsycInfo (via ProQuest)	(MAINSUBJECT.EXACT("Coronavirus") OR COVID19 OR COVID-19 OR "coronavirus disease 2019" OR SARS-CoV-2 OR "severe acute respiratory syndrome coronavirus 2" OR 2019-nCoV OR "novel coronavirus") AND (MAINSUBJECT.EXACT("Emerging Adulthood") OR MAINSUBJECT.EXACT("Students") OR MAINSUBJECT.EXACT.EXPLODE("College Students") OR "emerging adult*" OR "young adult*" OR student* OR universit* OR college* OR undergraduate*) AND (MAINSUBJECT.EXACT.EXPLODE("Hypertension") OR MAINSUBJECT.EXACT.EXPLODE("Heart Disorders") OR MAINSUBJECT.EXACT("Cerebrovascular Accidents") OR cardiovasc* OR heart OR hypertension OR "high blood pressure*" OR arrhythmia* OR myocar* OR coronar* OR ischem* OR stroke* OR cerebrovasc* OR valv* OR cardiomyopath* OR MAINSUBJECT.EXACT("Physical Activity") OR MAINSUBJECT.EXACT.EXPLODE("Exercise") OR MAINSUBJECT.EXACT.EXPLODE("Recreation") OR "physical activit*" OR exercis* OR recreation* OR sport* OR MAINSUBJECT.EXACT.EXPLODE("Tobacco Smoking") OR MAINSUBJECT.EXACT("Tobacco Use Disorder") OR MAINSUBJECT.EXACT.EXPLODE("Alcohol Drinking Patterns") OR MAINSUBJECT.EXACT.EXPLODE("Alcohol Use Disorder") OR tobacco OR alcohol* OR MAINSUBJECT.EXACT("Depression (Emotion)") OR MAINSUBJECT.EXACT("Major	n=951 (651+300)

	Depression") OR MAINSUBJECT.EXACT("Dysthymic Disorder") OR depress* OR dysthymi*)	
Sociological Abstracts	(COVID19 OR COVID-19 OR "coronavirus disease 2019" OR SARS-CoV-2 OR "severe acute respiratory syndrome coronavirus 2" OR 2019-nCoV OR "novel coronavirus") AND (MAINSUBJECT.EXACT("Young Adults") OR MAINSUBJECT.EXACT("Students") OR MAINSUBJECT.EXACT.EXPLODE("College Students") OR "emerging adult*" OR "young adult*" OR student* OR universit* OR college* OR undergraduate*) AND (MAINSUBJECT.EXACT("Blood Pressure") OR MAINSUBJECT.EXACT("Heart Diseases") OR cardiovasc* OR heart OR hypertension OR "high blood pressure*" OR arrhythmia* OR myocar* OR coronar* OR ischem* OR stroke* OR cerebrovasc* OR valv* OR cardiomyopath* OR MAINSUBJECT.EXACT("Physical Fitness") OR MAINSUBJECT.EXACT("Recreation") OR MAINSUBJECT.EXACT("Sports") OR MAINSUBJECT.EXACT("College Sports") OR "physical activit*" OR exercis* OR recreation* OR sport* OR MAINSUBJECT.EXACT("Smoking") OR MAINSUBJECT.EXACT("Drinking Behavior") OR MAINSUBJECT.EXACT("Alcohol Abuse") OR MAINSUBJECT.EXACT("Alcoholism") OR tobacco OR alcohol* OR MAINSUBJECT.EXACT("Depression (Psychology)") OR depress* OR dysthymi*)	n=610 (267+343)

Supplemental Table S2. Case reports and series (n=117) featuring cardiovascular conditions among emerging adults aged 18-25 with a COVID-19 context.

#	Authors (et al.)	Country	Age	Sex	Earliest reported time of known COVID-19 and/or symptoms	PCR results	IgG results	Comorbidities	CVD history	CV presentations	Treatments	Discharge status
**Emerging adults with reported COVID-19-related MIS-like hyperinflammatory syndromes (n=47)**												
**1**	Chau (n=4)	US	20	M	Admission	⊕	⊕	Previously healthy	N	Sinus tachycardia, severe left ventricular dysfunction/heart failure, mitral valve regurgitation, cardiogenic shock	Corticosteroids (methylprednisolone), anticoagulation	**Recovered**
			24	M	Unknown	⊖	⊕	Previously healthy	N	Atrial fibrillation, severe left ventricular dysfunction/heart failure, cardiogenic shock	Corticosteroids (methylprednisolone), anticoagulation	**Recovered**
			20	M	Admission	⊕	⊕	Previously healthy	N	Tachycardia, severe left ventricular dysfunction/heart failure, cardiogenic shock	Corticosteroids (methylprednisolone), anticoagulation	**Recovered**
			24	M	Admission	⊕	⊕	Alcohol abuse	N	Tachycardia, severe left ventricular dysfunction/heart failure, cardiogenic shock	Corticosteroids (methylprednisolone), anticoagulation	**Recovered**
**2**	Razavi	US	23	M	1 month prior	⊖	⊕	Hypertriglyceridemia, obesity	N	Tachycardia, hypotension, non-ST-elevation myocardial infarction	IVIG, methylprednisolone, aspirin, enoxaparin	**Recovered**
**3**	Morris (n=5)	US	21	M	Unknown	⊖	⊕	Obesity	N	Right ventricular dysfunction, mild mitral valve regurgitation	IVIG, corticosteroids, aspirin	**Recovered**
			22	F	Unknown	⊕	⊕	Previously healthy	N	Intermittent complete heart block	Corticosteroids, heparin	**Recovered**
			21	F	4 weeks prior	⊕	⊕	Obesity	N	Mild tricuspid and mitral valve regurgitation, pericardial effusion, left ventricular dysfunction	Corticosteroids, heparin, aspirin, dobutamine	**Recovered**
			21	M	Unknown	⊖	⊕	Previously healthy	N	Tachycardia	IVIG, corticosteroids, aspirin	**Recovered**
			25	F	Unknown	⊕	⊕	Previously healthy	N	Right ventricular dysfunction	IVIG, aspirin	**Recovered**
**4**	Kofman	US	25	F	Admission	⊕	⊕	Previously healthy	N	Cardiogenic shock, hypotension, pulmonary hypertension, ventricular dysfunction	IVIG, aspirin, anticoagulation	**Recovered**
**5**	Othenin-Girard	Switzerland	22	M	3 weeks prior	⊕	⊕	Previously healthy	N	Cardiogenic shock, myocarditis, ventricular dysfunction	IL-6 inhibitors, IVIG	**Recovered**
**6**	Vieira	Portugal	18	M	Admission	⊕	N/A	Previously healthy	N	Cardiogenic shock, myocarditis, mitral regurgitation	IVIG, methylprednisolone, aspirin, anticoagulation	**Recovered**
**7**	Cogan	Belgium	19	F	1 month prior	⊖	⊕	Previously healthy	N	Tachycardia, fulminant myocarditis	IL-6 inhibitors, IVIG, methylprednisolone	**Recovered**
**8**	Singh	US	18	M	Admission	N/A	N/A	Previously healthy	N	Ventricular dysfunction, cardiogenic shock, tachycardia, myocarditis	IVIG, anakinra, high-dose steroids	**Recovered**

**9**	Wojnowski	US	24	M	6 weeks prior	N/A	N/A	N/A	N/A	Tachycardia, ventricular dysfunction, myocarditis, non-ST-elevation myocardial infarction	Methylprednisolone, anticoagulation	**Recovered**
**10**	Nwachukwu	US	20	M	5 weeks prior	⊖	⊕	Beta-thalassemia	N	Tachycardia, cardiogenic shock	IVIG, corticosteroids, aspirin	**Recovered**
**11**	Bulut	Netherlands	20	M	5 weeks prior	⊕	⊕	Previously healthy	N	Tachycardia, ventricular dysfunction/heart failure, slight tricuspid valve insufficiency	IVIG, prednisone, aspirin	**Recovered**
**12**	MacDonald	US	18	M	1 month prior	⊕	⊕	N/A	N/A	Severe ventricular dysfunction	IVIG, steroids, anakinra	**Recovered**
**13**	Bonnet	France	19	M	1 month prior	⊕	N/A	N/A	N/A	Cardiogenic shock	Supportive care (no immunosuppressive therapy required)	**Recovered**
**14**	Carvalho	Brazil	21	F	Admission	⊕	N/A	N/A	N/A	Hypotension, ventricular dysfunction	IVIG, methylprednisolone	**Recovered**
**15**	Moghadam	France	21	M	Admission	⊖	⊕	Previously healthy	N	Hypotension, cardiomyopathy, heart failure	Anticoagulation	**Recovered**
**16**	Faller	Ireland	23	M	5 weeks prior	⊖	⊕	Previously healthy	N	Ventricular dysfunction/heart failure	Anticoagulation (heparin)	**Recovered**
**17**	Campoy	US	20	F	Second admission	⊖	⊕	Previously healthy	N	Tachycardia, pericardial effusion	IVIG, aspirin	**Recovered**
**18**	Bulathsinghala	US	23	M	5 weeks prior	N/A	N/A	N/A	N/A	Tachycardia, ventricular dysfunction, collapsible inferior vena cava, heart failure	IVIG, steroids, aspirin	**Recovered**
**19**	Aggarwal	US	21	M	Positive close contact 3 weeks prior	⊖	⊕	N/A	N/A	Tachycardia, myocarditis, ventricular dysfunction/heart failure	IVIG, anakinra, methylprednisolone, prednisone	**Recovered**
**20**	Szawarski	UK	20	M	6 weeks prior	N/A	N/A	N/A	N/A	Hypotension, left ventricular dysfunction	IVIG, methylprednisolone	**Recovered**
**21**	Al-Mashdali	Qatar	21	M	3 weeks prior	⊖	⊕	Previously healthy	N	Tachycardia, hypotension, pericardial effusion, ventricular dysfunction, suspected myocarditis	Supportive care and antibiotics (no immunosuppressive therapy required)	**Recovered**
**22**	Mittal	US	20	M	8 weeks prior	⊖	N/A	Previously healthy	N	Myocarditis, heart failure, tachycardia, hypotension, left ventricular dysfunction, small pericardial effusion mild pulmonic and tricuspid regurgitation	IVIG, methylprednisone, prednisone	**Recovered**
**23**	Ejaz	US	19	F	24 days prior	⊕	N/A	Previously healthy	N	Supraventricular tachycardia, hypotension, brief episode of paroxysmal atrial fibrillation	IVIG, solumedrol	**Recovered**
**24**	Rajendraprasad	US	19	M	6 weeks prior	N/A	N/A	Previously healthy	N	Tachycardia, hypotension, myopericarditis, left ventricular dysfunction	IVIG, aspirin, methylprednisolone, anakinra	**Recovered**
**25**	Pombo	Portugal	24	M	1 month prior	N/A	⊕	Previously healthy	N	Small pericardial effusion	Methylprednisolone, prednisolone	**Recovered**
**26**	Vujaklija Brajković	Croatia	22	M	4 weeks prior	⊖	⊕	Previously healthy	N	Tachycardia, biventricular dysfunction	IVIG, methylprednisolone, anticoagulation, aspirin	**Recovered**
**27**	Chug	US	25	M	6 weeks prior	⊖	⊕	N/A	N/A	Ventricular dysfunction/heart failure, cardiogenic shock	Methylprednisolone	**Recovered**

**28**	Nguyen	US	20	M	1 month prior	⊖	⊕	N/A	N/A	Shock, ventricular dysfunction/heart failure	Steroids, IVIG	**Recovered**
**29**	Dabas	India	22	M	Unknown	⊖	⊕	N/A	N/A	Tachycardia	Anticoagulation	**Recovered**
**30**	Ciochetto	US	21	M	Admission	⊕	N/A	Previously healthy	N	Tachycardia, ST and T wave abnormalities, left ventricular dysfunction	IVIG, aspirin, prednisone	**Recovered**
**31**	Ronit (n=2)	Denmark	19	M	4 weeks prior	⊖	N/A	Mild asthma	N	Hypotension, left ventricular dysfunction, cardiogenic shock	IVIG	**Recovered**
			23	F	7 weeks prior	N/A	N/A	N/A	N/A	Tachycardia, T wave inversion, myocarditis	IVIG, methylprednisolone, aspirin	**Recovered**
**32**	Malik	US	19	F	N/A	N/A	⊕	CHD (Ebstein anomaly); orthotopic heart transplant 13 years ago	Ebstein anomaly; heart transplan t	13 years post-heart transplant with high-grade acute cellular rejection; cardiac tamponade and multi-organ failure due to pulmonary artery origin massive hemomediastinum, coagulopathy	N/A	**Died**
**33**	Hékimian (n=3)	France	19	F	Unknown	⊖	⊕	Previously healthy	N	Hypotension, tachycardia, left ventricular dysfunction	Only supportive care	**Recovered**
			19	M	Unknown	⊖	⊕	Previously healthy	N	Hypotension, tachycardia, left ventricular dysfunction, diffuse edema	Only supportive care	**Recovered**
			25	F	Unknown	⊖	⊕	Previously healthy	N	Tachycardia, negative T waves in D2-D3-aVF, diffuse edema, intramural necrosis, left ventricular dysfunction	Only supportive care	**Recovered**
**34**	Bastug	Turkey	18	F	2 months prior	⊖	⊕	N/A	N/A	Hypotension, tachycardia, left ventricular dysfunction, diffuse edema	Methylprednisolone, IVIG, anticoagulation	**Recovered**
**35**	Salzman (n=3)	US	20	F	Admission	⊕	⊕	Asthma	N	Cardiogenic shock, tachycardia, hypotension, left ventricular dysfunction	IVIG, methylprednisolone, anticoagulation, remdesivir	**Recovered**
			18	M	6 weeks prior	⊖	⊕	Asthma	N	Hypotension, tachycardia, left ventricular dysfunction	IVIG, methylprednisolone, anakinra	**Recovered**
			23	M	1 month prior	⊕	N/A	Asthma, obesity	N	Ventricular dysfunction, tachycardia, cardiogenic shock, hypotension	IVIG, methylprednisolone	**Died**

Note: All patients received some form of 'supportive care', including supplemental oxygen, antibiotics, vasopressors, inotropes, fluids, etc.

#	Authors (et al.)	Country	Age	Sex	Earliest reported time of known COVID-19 and/or symptoms	PCR results	IgG results	Comorbidities	CVD history	Co-occurring conditions	CV presentations	CV treatments	Discharge status
**Emerging adults without reported COVID-19-related MIS-like hyperinflammatory syndromes (n=76)**													
**36**	Molina	US	23	M	Admission	⊕	N/A	Nitrous oxide inhalant abuse, ?antiphospholipid antibody syndrome	N	Hyperhomocystei nemia, ?antiphospholipid antibody syndrome	Tachycardia, hypotension, right bundle branch block, right heart strain, saddle pulmonary embolism, left internal jugular vein thrombosis	Tissue plasminogen activator (alteplase)	**Recovered**
**37**	Rodrig uez	US	21	M	3 days prior	⊕	N/A	Substance abuse (cocaine, methamphetamin	N	Parainfluenza 4 virus co-infection	Tachycardia	N/A	**Recovered**

								es), smoking history					
**38**	Cardoso	US	18	F	2 days prior	⊕	N/A	Autism spectrum disorder, panic disorder	N	Systemic lupus erythematosus, acute respiratory distress syndrome, pleural effusion	Tachycardia, hypotension, cardiac arrest, pericardial effusion (likely myopericarditis), cardiac tamponade, left ventricular dysfunction/heart failure, multiple deep vein thromboses	Pericardiocentesis, anticoagulation	**Died**
**39**	Wongkittichote	US	23	F	3 days prior	⊕	N/A	Long-chain fatty-acyl CoA dehydrogenase deficiency	Long QT with self-limited torsade de pointes during pregnancy	Rhabdomyolysis, bilateral pleural effusions, bacteremia, acute respiratory failure, ischemic nonocclusive enteritis	Prolonged QT interval, hypotension, right ventricular dysfunction, right ventricular hypokinesis, left ventricular dysfunction, acute cardiomyopathy, venous thromboembolism, combined cardiogenic and septic shock	Enoxaparin, alteplase, vasopressin, epinephrine, norepinephrine, ECMO	**Died**
**40**	Jeantin	France	18	F	8 days prior	⊕	N/A	Previously healthy	N	Acute kidney injury, bilateral proximal paraparesia of lower limbs, acute respiratory distress syndrome, suspected Guillain-Barre syndrome	Tachycardia, hypotension, ventricular dysfunction/heart failure with global hypokinesia, cardiogenic shock, myocarditis	Norepinephrine, dobutamine, nitric oxide, dexamethasone, ECMO	At time of publication: remains hospitalized under veno-venous ECMO
**41**	Soquet	France	22	F	Unknown	⊕	⊖	Sub-diaphragmatic mass related to Epstein-Barr virus-induced lymphoma	Successful heart transplant for giant cell myocarditis, subsequent pericardial constriction treated with pericardiectomy	Right-sided pleural effusion, severe pulmonary COVID-19 infection	Biventricular dysfunction/heart failure, chronic rejection of heart transplant	Veno-arterial ECMO, low-dose dobutamine, methylprednisolone and rabbit anti-thymocyte globulin, heart re-transplantation	**Recovered**
**42**	Garau	Belgium	18	F	1 day prior	⊕	N/A	Previously healthy	N	Metabolic acidosis, acute kidney injury, pulmonary edema, acute respiratory distress syndrome, bilateral pleural effusion, rhabdomyolysis	Tachycardia, hypotension, ST elevation and PR depression, left ventricular wall thickening, myocardial edema, mild inferior pericardial effusion, severe biventricular dysfunction/heart failure, ventricular hypokinesia, possible fulminant myocarditis, cardiogenic shock, severe coagulopathy	Dobutamine, vasopressors, veno-arterial ECMO, intra-aortic balloon pump, IVIG, methylprednisone	**Recovered**

**43**	Crippa	Switzerland	24	M	1 day prior	⊕	⊕	Previously healthy	N	N/A	Ischemic stroke, vascular endothelial dysfunction, endotheliitis	Aspirin, enoxaparin	**Recovered**
**44**	Beşler	Turkey	20	M	Unknown	⊕	N/A	Previously healthy	N	COVID-19 pneumonia	Tachycardia, myocarditis	Colchicine	**Recovered**
**45**	Garot	France	18	M	Unknown	⊕	N/A	Previously healthy	N	COVID-19 pneumonia, acute respiratory distress	Tachycardia, hypotension, mild left ventricular enlargement, diffuse hypokinesis (heart failure), mild pericardial effusion, fulminant myocarditis	Norepinephrine, bisoprolol (at discharge), angiotensin-converting enzyme inhibitor (at discharge)	**Recovered**
**46**	Kim	South Korea	21	F	Unknown	⊕	N/A	N/A	N/A	N/A	Non-specific intraventricular conduction delay, premature ventricular complexes, severe left ventricular systolic dysfunction, myocarditis	N/A	N/A
**47**	Alizade hasl	Iran	24	M	3 weeks after discharge from COVID-19 infection	⊕	N/A	Rheumatic heart disease	Rheumatic heart disease with history of mechanical mitral valve replacement	Bacteremia (Staphylococcus aureus)	Tachycardia, infective endocarditis (vegetations on posterior mitral valve leaflet)	Corticosteroids and antibiotics	**Recovered**
**48**	Fatehi	Iran	18	M	1 week prior	⊕	N/A	Previously healthy	N	Quadriparesis, respiratory distress, bilateral pleural effusion	Ischemic stroke	N/A	N/A
**49**	Krishnan (n=2)	US	25	F	Unknown	⊕	N/A	Down syndrome, obstructive sleep apnea	Unrepaired atrioventricular septal defect	Dehydration	Hypotension	Only supportive, antibiotics, sarilumab (clinical trial)	**Recovered**
		US	21	M	Unknown	⊕	N/A	Down syndrome, obstructive sleep apnea	Unrepaired atrioventricular septal defect, pulmonary hypertension	SARS-CoV-2 pneumonia-associated respiratory failure	Prolonged QTc interval (antibiotic side effect)	Ambrisentan and tadalafil (for pulmonary hypertension), inhaled nitrous oxide, tocilizumab	**Recovered**
**50**	Carneiro	US	24	M	Unknown	⊕	N/A	Diabetes, dyslipidemia, obesity, cocaine use	N	N/A	Cardioembolic stroke leading to asymptomatic petechial hemorrhagic transformation, atrial fibrillation	tPA (for stroke)	**Recovered**
**51**	Calhoun	US	23	M	3 days prior	⊕	N/A	Mild intermittent asthma	N	Bilateral pleural effusion and pulmonary edema	Tachycardia, diffuse ST elevation, hypotension, biventricular systolic dysfunction and global left ventricular hypokinesis (heart failure), cardiogenic shock, fulminant myocarditis	Dobutamine, dexamethasone, anticoagulation (at discharge)	**Recovered**

**52**	Choudhary	US	24	M	Several days prior	⊕	N/A	Previously healthy	N	N/A	Tachycardia, myocarditis, biventricular systolic dysfunction (acute decompensated heart failure)	Furosemide, metoprolol, convalescent plasma, remdesivir, dexamethasone, Lovenox, norepinephrine, discharge medications (furosemide, metoprolol succinate, lisinopril, aspirin, atorvastatin, Rivaroxaban)	**Recovered**
**53**	Volis	Israel	21	M	20 days prior	⊕	N/A	Previously healthy, social smoker	N	N/A	Tachycardia, subclinical acute myocarditis	Only supportive	**Recovered**
**54**	Laleh Far	Iran	23	F	Unknown	⊕	N/A	Idiopathic hypereosiniphilic syndrome	N	N/A	Tachycardia, moderate left ventricular systolic dysfunction and increased wall thickness, myocardial inflammation with pericardial effusion	Colchicine, naproxen, methylprednisolone pulse then oral prednisolone	**Recovered**
**55**	Venditti	France	25	F	1 month prior	N/A	⊕	N/A	N/A	N/A	Multiple acute and subacute ischaemic cortical strokes (caused by Susac syndrome), arteriolar occlusions, focal retinal arteriolar vasculitis	Methylprednisolone then oral prednisone, immunoglobulin therapy, aspirin	**Recovered**
**56**	Bhattarai	US	19	F	4 days prior	⊕	N/A	Reynaud's phenomenon	N	Cerebral edema, Addison's disease (initial presentation)	Tachycardia, hypotension	Norepinephrine, hydrocortisone	**Recovered**
**57**	Bozan	Turkey	23	M	8 days prior	⊕	N/A	N/A	N	Spontaneous pneumothorax following pneumomediastinum, pleural effusion	Tachycardia	Only supportive	**Recovered**
**58**	O'Connor	UK	23	M	Self-resolved COVID-19 in November 2020	N/A	N/A	N/A	N/A	Uncomplicated appendicitis, mesenteric lymphadenopathy, bilateral lower lobe consolidation and ground-glass opacities (suspicious of COVID-19)	Hypotension, myocarditis, left ventricular systolic dysfunction/heart failure	Vasopressor support, cardioprotective medication	**Recovered**

**59**	Recalcati	Italy	19	F	4 days prior	⊕	N/A	N/A	N/A	N/A	Tachycardia, diffuse ST elevation, acute myoperidarditis	Colchicine, low molecular weight heparin, aspirin, pantoprazole	**Recovered**
**60**	Dahou	UK	24	F	A few days prior	⊕	N/A	Previously healthy	N	Systemic lupus erythematosus (new diagnosis)	Hypertension, hemolytic anemia, thrombocytopenia	Pulse steroid therapy	**Recovered**
**61**	Sheha	Egypt	22	F	A few days prior	⊕	N/A	Previous splenectomy for anemia and recurring bony aches thought to be rheumatic fever	N	Sickle cell disease (new diagnosis) leading to vaso-occlusive crisis, moderate COVID-19 pneumonia, minimal right pleural effusion suggestive of multilobar pneumonia	Tachycardia, impending heart failure, autoimmune hemolytic anemia and thrombocytopenia	Two packs of red blood cells, anticoagulation, corticosteroids	**Recovered**
**62**	Singh	US	24	M	Unknown	⊕	N/A	Type 1 diabetes mellitus, hyperlipidemia	Hyperlipidemia	Diabetic ketoacidosis, sepsis, acute respiratory distress syndrome, COVID-19 pneumonia	Hypertension and tachycardia, deteriorated to hypotension and bradycardia	Only supportive and antibiotics/antifungals	**Died**
**63**	Han	China	20	M	3 days prior	⊕	N/A	Acute B lymphoblastic leukemia	N	Acute graft-versus-host disease after haploidentical transplantation, septic shock, multiple organ failure	Tachycardia, coagulation dysfunction, multi-organ failure	Methylprednisolone (presumably for acute graft-versus-host disease or leukemia), chemotherapy (for his leukemia), norepinephrine	**Died**
**64**	Strause	US	24	M	2 weeks prior	⊕	N/A	Previously healthy	N	N/A	Bilateral cerebral infarctions (stroke), bilateral aneurysmal lenticulostriate vasculopathy	Aspirin	**Recovered**
**65**	Merdad	Saudi Arabia	23	M	2-3 days prior	⊕	N/A	Previously healthy	N	Perforated duodenal ulcer, lower chest ground-glass opacity, multi-organ failure	Tachycardia, evolving cardiac arrhythmias, internal jugular vein thrombosis, multi-organ failure	Heparin	**Died**
**66**	Bunawan	Indonesia	22	F	8 days prior	⊕	⊕	Previously healthy	N	Right-sided COVID-19 pneumonia	Tachycardia	N/A	**Recovered**
**67**	Gaglani	US	18	M	Unknown	⊕	N/A	Asthma, obstructive sleep apnea, super morbid obesity	N	Acute respiratory distress syndrome	ST-elevation myocardial infarction, high endogenous cardiac output, shock	Thrombolytics, beta-blockers, catecholamine, vasopressin,	**Recovered**

												angiotensin II support	
**68**	Pasqualetto	Italy	22	M	Admission	⊕	N/A	CHD (partial anomalous pulmonary venous drainage, PAPVD), previously asymptomatic	CHD (PAPVD), previously asymptomatic	COVID-19 pneumonia	Right bundle branch block, pulmonary hypertension	Referred for to an adult congenital heart disease centre for surgical repair	**Recovered**
**69**	Rashed	US	22	M	Admission	⊕	N/A	Previously healthy	N	N/A	Hypertension, inferior and infero-lateral wall hypokinesis, ST-elevation myocardial infarction, proximal left circumflex artery stenosis (thrombotic occlusion)	Aspiration thrombectomy, Onyx 2.5x26 stent	**Recovered**
**70**	Freeman	Unknown	24	M	Unknown	N/A	N/A	Morbid obesity	N	?Propofol-related infusion syndrome, acute respiratory distress syndrome secondary to COVID-19 pneumonia, renal failure	Bradycardia, hypotension, sudden cardiac arrest	Vasopressor support, cardiopulmonary resuscitative efforts	**Died**
**71**	Ouzts	US	18	F	12 days prior	⊕	N/A	N/A	N/A	N/A	Myocardial injury	N/A	**Recovered**
**72**	Edwards	US	21	F	1 day prior	⊕	N/A	Migraines	N	Hyperthyroidism (acute severe thyrotoxicosis)	Tachycardia, hypotension	Propranolol	**Recovered**
**73**	Munoz	US	18	M	~1 month prior	⊕	N/A	Obesity	N	N/A	Left ventricular dysfunction/heart failure, left ventricle thrombus, myocarditis	Heparin, warfarin, standard heart failure therapy (losartan, carvedilol, and spironolactone)	**Recovered**
**74**	Eid	United Arab Emirates	20	M	15 days prior	⊕	N/A	N/A	N/A	P. falciparum malaria co-infection	Tachycardia	N/A	**Recovered**
**75**	Clerico	Italy	22	M	1 week prior	⊕	⊖	Diffuse large B-cell lymphoma (DLBCL), congenital heart disease - congenital hypertrophic cardiomyopathy (heart transplant in August 2019)	CHD (congenital hypertrophic cardiomyopathy, heart transplant in August 2019)	Post-transplant lymphoproliferative disorder (EBV-positive monomorphic DLBCL-type PTLD), bilateral lung ground-glass infiltrates	Hypotension, small circumferential pericardial effusion	Fluid resuscitation	**Recovered**
**76**	Hasnie	US	18	M	6 weeks prior	N/A	N/A	N/A	N/A	N/A	Sinus bradycardia without significant ST changes, abnormal late gadolinium	Supportive therapy	**Recovered**

											enhancement in the inferior and inferolateral walls in the distal portion of the left ventricle (suspected myocarditis)		
**77**	Sikandar	US	23	F	Unknown	⊖	⊕	Obesity	N	Acute kidney injury and shock liver secondary to hypoperfusion from cardiogenic shock	Tachycardia, ST-elevation myocardial infarction, laminated thrombus in the left anterior descending (LAD) coronary artery, cardiogenic shock, left ventricular dysfunction/heart failure	Aspirin, balloon angioplasty of the proximal LAD followed by aspiration thrombectomy (no reflow was observed), intra-coronary adenosine and nicardipine (some improvement), repeat aspiration thrombectomy (significant improvement), vasopressor support and intra-aortic balloon insertion (cardiogenic shock), epinephrine, norepinephrine, veno-arterial ECMO	**Recovered**
**78**	Hekmatikar	Iran	20	F	2 days prior	N/A	N/A	Overweight	N	Lung involvement	Tachycardia	Exercise protocol	**Recovered**
**79**	Guendouz	France	19	F	1 week prior	⊕	N/A	Migraine, morbid obesity	N	First tonic-clonic seizure, bilateral ground-glass opacities affecting pulmonary parenchyma	Thrombosis of the superior sagittal sinus, frontal cortical veins, and right sigmoid sinus	Anticoagulation therapy (low-molecular-weight heparin, vitamin K antagonist)	**Recovered**
**80**	Bozorgmehr	Iran	21	M	1 week prior	⊕	N/A	Previously healthy	N	Bilateral ground-glass opacities and multifocal consolidation, respiratory failure and multi-organ damage	Tachycardia, multi-organ damage	Supportive therapy	**Died**
**81**	Hirschbaum	US	21	M	3 days prior	⊕	N/A	Obesity	N	Respiratory failure	Tachycardia, right heart strain as well as S wave in lead I, Q wave and inverted T wave in lead III (S1Q3T3), recurrent massive pulmonary embolism, hypotension, cardiac arrest,	Catheter-directed thrombolysis (CDT), cardiopulmonary resuscitation and electrical shock,	**Recovered**

											deep venous thrombus of the right femoral vein and right popliteal vein	vasopressor support, veno-arterial ECMO, repeat CDT, inferior vena cava filter, unfractionated to low-molecular-weight heparin, rivaroxaban	
**82**	Hussein	Saudi Arabia	21	M	3 weeks prior	⊕	⊕	Steroid-dependent nephrotic syndrome, morbid obesity, hypertension, pulmonary embolism, type 2 protein S deficiency	Hypertension, pulmonary embolism	Sepsis, acute kidney injury (requiring renal transplant therapy), respiratory failure, abdominal compartment syndrome, intestinal infarction with pneumatosis intestinalis and pneumoperitoneum, short bowel syndrome	Hypotension, portal vein thrombosis (propagated to involve the spleno-mesenteric, intrahepatic and the right hepatic veins)	Intravenous fluids, anticoagulation (heparin), catheter-directed thrombectomy followed by a transjugular intrahepatic portosystemic shunt insertion, thrombolytics, lifetime anticoagulation	**Recovered**
**83**	Doğan	Turkey	22	M	2 weeks prior	N/A	N/A	Previously healthy	N	Bilateral ground-glass opacities (COVID-19 pneumonia)	Tachycardia, mild enlargement in the right heart chambers, mild-to-moderate tricuspid regurgitation, saddle pulmonary embolism	Enoxaparin	**Recovered**
**84**	Bhasin	US	23	M	Unknown	⊕	⊖	Stage IIIB Hodgkin's lymphoma	N	N/A	Tachycardia, hypotension, right bundle branch block, reversible complete heart block, acute myocarditis, moderate concentric left ventricular hypertrophy, borderline dilation of the right ventricle, mild RV hypertrophy, heart failure, trace pericardial effusion	Fluid resuscitation, intravenous norepinephrine, transvenous pacemaker, milrinone	**Recovered**
**85**	Abdullah	Germany	18	F	5 days prior	⊕	N/A	Ulcerative colitis	N	N/A	Tachycardia	N/A	**Recovered**
**86**	Ghosh	India	20	M	8 days prior	⊕	N/A	Previously healthy	N	Acute motor axonal neuropathy (AMAN)	Tachycardia, hypertension, postural hypotension, dysautonomia (loss of heart rate variability and reflex blood pressure changes)	IVIG (for AMAN)	**Recovered**
**87**	Long	US	23	M	Admission	⊕	N/A	Smoking history	N	Left hydropneumothorax with moderate to large	Tachycardia, hypotension, hemorrhagic shock	Tube thoracostomy, massive	**Recovered**

										pneumothorax component, moderate left-sided effusion, mild associated right mediastinal shift		transfusion protocol	
**88**	Senevir atna	Sri Lanka	19	M	Admission	⊕	⊕	Previously healthy	N	Open hairline non-displaced fracture of the right tibia (road traffic accident), pneumonia	Tachycardia, fat emboli	Enoxaparin	**Recovered**
**89**	Flower	UK	22	F	8 weeks prior	⊕	N/A	Sickle trait	N/A	Hemophagocytic lymphohistiocyto sis, mild bibasilar subpleural ground-glass changes, pelvic free fluid, peritoneal enhancement, hepatosplenomeg aly	Tachycardia, hypotension	Inotropic support	**Recovered**
**90**	Simpso n	US	24	F	Admission	⊕	⊖	Focal segmental glomerulosclerosi s	N/A	Hemolytic uremic syndrome, acute kidney injury, focal seizures, right hemiparesis	Tachycardia	N/A	**Recovered**
**91**	Pąchals ka	Poland	23	M	Admission	⊕	N/A	N/A	N/A	N/A	Right hemisphere stroke	Anodal transcranial direct current stimulation	**Recovered**
**92**	Aikawa	Japan	18	M	3 months prior	⊕	N/A	N/A	N/A	N/A	Endocarditis complicating myocardial fibrosis	N/A	**Recovered**
**93**	Marcin kiewicz	Poland	20	M	6 weeks prior	⊖	⊕	N	N	N/A	Fulminant myocarditis, ventricular dysfunction/acute heart failure	Norepinephrine, dobutamine, intra-aortic balloon pump (mild improvement), veno-arterial ECMO	**Recovered**
**94**	Al-Kaf	Saudi Arabia	21	M	2 days prior	⊕	N/A	Down syndrome, congenital tracheobronchom alacia (successfully treated with surgery)	N	Severe hypothyroidism, acute respiratory distress syndrome	Hypotension, pericardial effusion, cardiac tamponade	Pericardiocentesis , heparin	**Recovered**
**95**	Krishna	UK	22	M	4 days prior	⊕	N/A	Obesity, hypothyroidism	N	Extensive consolidation in the right mid-to-	Acute anterior cerebral artery territory infarct (stroke), pulmonary embolism,	Aspirin, low-molecular-weight heparin,	**Died**

										lower lung zones (COVID-19 pneumonia), rhabdomyolysis, acute hemorrhagic pancreatitis, steatohepatitis, thyroiditis, acute tubular injury of the kidneys, multi-organ failure secondary to disseminated mucormycosis	pericarditis, cardiac tamponade, vasoplegic shock, thrombo-emboli in the lungs, brain, pharynx, nasal mucosa, and trachea	vasopressor support, emergency pericardiocentesis	
**96**	Alam	US	21	M	2 weeks prior	N/A	N/A	Previously healthy	N	N/A	Sinus bradycardia with ST elevation in inferior leads, hypokinesis of inferior wall of the left ventricle consistent with acute myocarditis, ventricular dysfunction	Aspirin, ticagrelor, atorvastatin, bivalirudin, carvediol, captopril	**Recovered**
**97**	O'Sullivan	Ireland	22	F	3 weeks prior	⊕	N/A	Previously healthy	N	N/A	Tachycardia, postural orthostatic tachycardia syndrome	Ivabradine	**Recovered**
**98**	Hedayat	Iran	24	M	5 days prior	⊕	N/A	Previously healthy	N	Bilateral ground-glass peripheral densities in lower lobes	Tachycardia, myocarditis	Metoral	**Recovered**
**99**	Kenniff	US	22	F	3-4 weeks prior	⊕	N/A	Ehlers-Danlos syndrome	N	Diffuse myoclonic disorder	Bradycardia	N/A	**Recovered**
**100**	Arandela (n=2)	US	25	F	2 days prior	⊕	N/A	Hypertension, obesity, marijuana use	Hypertension	N/A	Reversible cerebral vasoconstriction syndrome, hypertension, acute ischemic bilateral occipital strokes, diffuse vasospasm	Instructed to stop marijuana use, nicardipine	**Recovered**
		US	21	M	>30 days prior	⊕	N/A	Previously healthy	N	Liver/pancreas/renal/respiratory failure, encephalopathy	Reversible cerebral vasoconstriction syndrome, diffuse intracranial vascular abnormalities concerning for vasospasm or vasculitis, cardiogenic shock, ventricular dysfunction/heart failure	Unclear	At time of publication: currently admitted, extubated in ICU, to be downgraded soon
**101**	Petracek	US	19	M	3 days prior	⊕	⊕	Gilbert syndrome, asthma, allergies, several food intolerances	N	N/A	Tachycardia, bradycardia, postural orthostatic tachycardia syndrome	Methylphenidate, compression garments, increased sodium intake	Myalgic encephalomyelitis or chronic fatigue syndrome

**102**	Elikowski (n=2)	Poland	24	M	Unknown	⊕	N/A	Previously healthy	N	N/A	Bradycardia	N/A	**Recovered**
**103**	Heidarpour	Poland	20	F	Unknown	⊕	N/A	Obesity	N	Moderate pulmonary involvement based on computed tomography	Bradycardia	N/A	**Recovered**
		Iran	22	M	20 days prior	N/A	N/A	Opium abuse	N	Severe hypocalcemia, rhabdomyolysis, multifocal peribronchial consolidation and diffuse ground-glass opacities, myoclonic-like seizure, respiratory arrest	Prolonged QT interval, cardiac arrest	Cardiopulmonary resuscitation	**Died**
**104**	Grisanti	Italy	18	N/A	Unknown	N/A	N/A	Previously healthy	N	N/A	Ischemic stroke	N/A	N/A
**105**	Croci	Italy	18	M	16 days prior	⊕	N/A	Previously healthy	N	Severe right pneumothorax with pneumatocele, pulmonary superinfections, acute respiratory distress syndrome	Severe pulmonary hypertension, systolic biventricular dysfunction, unexpected thrombosis	Anticoagulation (direct thrombin inhibitors)	**Recovered**
**106**	Mitchell (n=2)	US	19	M	Admission	⊕	N/A	Obesity	N	N/A	Pulmonary embolism	Anticoagulation (low-molecular-weight heparin)	**Recovered**
		US	21	M	Admission	⊕	N/A	Previously healthy	N	N/A	Pulmonary emboli (bilateral)	Anticoagulation (low-molecular-weight heparin)	**Recovered**
**107**	Sawalha	US	21	F	3 weeks prior	⊕	N/A	Previously healthy	N	N/A	Bradycardia, narrow QRS complexes and premature atrial complexes	Atropine, theophylline	**Recovered**

#	Authors (et al.)	Country	Age	Sex	Earliest reported time of known COVID-19 and/or symptoms	PCR results	IgG results	Comorbidities	CVD history	CV presentations	Discharge status
**Miscellaneous cases (n=5)**											
**108**	Quinlivan (n=5)	UK	18	M	December 2020	⊕	N/A	Duchenne muscular dystrophy, obesity	N/A	N/A	**Recovered**
			18	M	November 2020	⊕	N/A	Duchenne muscular dystrophy, cardiomyopathy	Cardiomyopathy	N/A	**Recovered**
			19	M	November 2020	⊕	N/A	Duchenne muscular dystrophy, severe	Severe dilated cardiomyopathy	N/A	**Recovered**

								dilated cardiomyopathy			
			20	M	October 2020	⊕	N/A	Duchenne muscular dystrophy, scoliosis, moderate-severe dilated cardiomyopathy	Moderate-severe dilated cardiomyopathy	N/A	Recovered
			23	M	Unknown	⊕	N/A	Duchenne muscular dystrophy, severe autism, mild scoliosis, moderately severe cardiomyopathy	Moderately severe cardiomyopathy	N/A	Recovered

#	Authors (et al.)	Country	Age	Sex	Study aim	Pertinent results	Discharge status
**Emerging adults with cardiovascular conditions indirectly related to COVID-19 (i.e. impacts on cardiovascular care) (n=9)**							
**109**	Kandori	Japan	24	M	To share the experience of resuscitating a suspected COVID-19 patient (24-year-old male) after out-of-hospital cardiac arrest, the authors' consideration of extracorporeal cardiopulmonary resuscitation (E-CPR), and the various challenges created by this decision.	The patient's heartbeat resumed after 54 minutes without E-CPR, and he was extubated four days after hospitalization. The authors recommend not performing E-CPR on patients suspected to have COVID-19.	**Recovered**
**110**	Warraich	UK	19	M	To describe the case of a 19-year-old male admitted to the hospital with a 2-week history of continuous cough and a 1-day history of acute onset unsteadiness and hiccups initially suspected to be COVID-19.	Brain MRI confirmed a small left acute and subacute lateral medullary infarction, and a chest X-ray suggested aspiration pneumonia with right lower lobe collapse (a distinctive case of **posterior circulation stroke**).	**Recovered**
**111**	Cruz-Utrilla	Spain	21	M	To describe the case of a 21-year-old male with a previous history of stage I sarcoidosis who was admitted to the emergency department for 2-week exertional dyspnea and bilateral leg edema.	The patient was diagnosed with stage III pulmonary sarcoidosis, pulmonary hypertension, and pressure overload-induced right ventricular remodeling and was treated accordingly; he was discharged ten days later. It was reported that the patient decided not to consult previously due to the local COVID-19 lockdown.	Hospitalized
**112**	Cankay	Turkey	18	F	To describe the case of an 18-year-old female with no history of medical or psychiatric diagnoses who was referred to the emergency department with symptoms of agitation, aggression, and left arm numbness during the COVID-19 pandemic.	The patient believed that she and her parents were exposed to COVID-19 in the emergency department, so they refused an MRI and left. She was re-referred five hours later with exacerbated symptoms. Cranial MRI and MRI angiography revealed acute infarct areas in the basal ganglia and periventricular white matter (**stroke**); the patient was treated with aspirin and klopidogrel.	Neurological assessment
**113**	Toth	UK	20	M	To describe the case of a 20-year-old male with a previous bicuspid aortic valve and aortic coarctation repair who presented with a 4-week history of vomiting, diarrhea, low-grade fever, productive cough, and progressive breathlessness (belated presentation) during the COVID-19 pandemic.	Electrocardiograms revealed first-degree atrioventricular block with widespread ischemia, severe aortic regurgitation, a large aortic valve vegetation, and aortic root abscess, later developing complete heart block and asystole.	**?Died**
**114**	Atuaka	US	19	M	To describe the case of a 19-year-old male with no significant past medical history presenting to the emergency department in July 2020 with a persistent	The patient's diagnosis of Coxsackie A myocarditis was clouded by the medical team's strong suspicion of COVID-19 pneumonia (which was initially treated with azithromycin and doxycycline), leading to readmission 6 days later.	**Recovered**

					fever for 10 days, suspected to have COVID-19 pneumonia.		
**115**	Greenfeld	Israel	19	M	To describe the case of a 19-year-old male diagnosed concomitantly with extensive thromboses (including two intracardiac masses and Budd-Chiari syndrome) as well as acute myeloid leukemia during the COVID-19 pandemic.	The patient was diagnosed with AML and multiple thromboses (two intracardiac thrombi, an inferior vena cava thrombosis, a pulmonary embolus, a hepatic vein thrombosis, and splenic infarcts). The authors suggested that the uncertainty in April 2020 regarding COVID-19 may have caused a delay in this patient seeking medical evaluation and contributed to this ‘catastrophic’ presentation.	**Recovered**
**116**	Daliri	Iran	18	F	To describe the case of an 18-year-old female who was hospitalized due to the poisoning aluminum phosphate during the COVID-19 pandemic.	At the time of arrival, the patient's ejection fraction (EF) was less than 5%; ECMO was weaned on day 5 when the EF was 55%. Following, the general and pulmonary status of the patient deteriorated when she was believed to have contracted COVID-19, resulting in additional therapeutic measures and discharge after 45 days.	**Recovered**
**117**	Balfe	Ireland	18	F	To describe the case of an 18-year-old female with newly presenting severe mitral valve stenosis during the peak of the COVID-19 pandemic.	The patient's condition was worsened by the IV fluids she was administered under the presumption that she had COVID-19; the true diagnosis was rheumatic mitral stenosis with accompanying pulmonary hypertension, eventually requiring intubation, vasopressor support, and percutaneous mitral balloon commissurotomy.	**Recovered**

Supplemental Table S3. Non-case studies (eg, observational studies and reviews) featuring cardiovascular conditions among emerging adult populations with a COVID-19 context (n=28).

#	Authors (et al.); **country**	Study aim	Population of interest, N	Age (years); % male	Methods	Timing of imaging	Pertinent results	Follow-up	Definitions	Prevalence estimates	Illness severity of participants
**Studies investigating myocarditis and/or other cardiac abnormalities (n=13)**											
Sample size (N) <100											
**118**	Vago; **Hungary**	To describe the cMRI results of elite athletes recently recovered from COVID-19 with mild to moderate symptoms.	Elite athletes, N=12	Median age: 23 (20-23); 16.7% male	**Cross-sectional study**: Professional, elite athletes after recovering from COVID-19 were referred for cMRI examination before returning to high levels of athletic performance. Patients underwent laboratory testing on the day of the cMRI examination.	Median time to imaging after testing positive for COVID-19: 17 days (17-19)	**cMRI**: No athlete showed myocardial or pericardial edema or pathological late gadolinium enhancement.	N/A	N/A	**Cardiac abnormalities (myocardial or pericardial edema or pathological LGE)**: 0% (0/12)	10 (83.3%) mild/moderate; 2 (16.7%) asymptomatic
**119**	Gervasi; **Italy**	To understand which investigations are useful in a screening protocol aimed at protecting health but also avoiding inappropriate examinations.	Student athletes, N=30 (18 COVID-19+ and 12 COVID-19-)	Median age: COVID-19+: 22 (range: 21-27); COVID-19-: 25 (range: 19.5-26.5); 100% male	**Cross-sectional study**: Screening measured performed: personal history, antigen swabs, blood tests, spirometry, resting/stress-test ECG with oxygen saturation monitoring, echocardiogram, Holter and chest CT. The findings were compared with prior data from the same subjects before infection and with data from SARS-CoV-2-negative players.	No participants complained of COVID-19-related and/or cardiac symptoms in the past 3 months	**ECG**: Resting ECG was normal (substantially the same) before and after the infection in all players, with some showing changes compatible with the 'athlete's heart', such as sinus bradycardia. Stress-test ECG showed rare, isolated ventricular premature beats (VPB) in one case (5.6%) and rare, isolated supraventricular premature beats (SVPB) in one case (5.6%) not present before infection. **cMRI** (performed in one athlete with increased cardiac troponin): No abnormal findings.	N/A	N/A	**Rare, isolated VPB**: 5.6% (1/18); r**are, isolated SVPB**: 5.6% (1/18); **significant CV abnormalities**: 0% (0/18)	12 (66.7%) mild (66.7%); 6 (33.3%) asymptomatic
**120**	Rajpal; **US**	To investigate the use of cMRI in competitive athletes recovered from COVID-19 to detect myocardial inflammation that would identify high-risk athletes for return to competitive play.	Student athletes, N=26	Mean age: 19.5; 57.7% male	**Cross-sectional study**: A comprehensive cMRI examination included cine, T1 and T2 mapping, extracellular volume fraction, and late gadolinium enhancement (LGE) on a 1.5-T scanner in all competitive athletes referred to the sports medicine clinic after testing positive for COVID-19. ECG, serum troponin I, and transthoracic echocardiogram were performed on day of cMRI.	Imaging was performed after recommended quarantine (11-53 days)	**ECG**: No diagnostic ST/T wave changes on electrocardiogram. **Echocardiogram**: Ventricular volumes and function were within the normal range in all athletes. **cMRI**: 4 athletes (15%; all male individuals) had cMRI findings consistent with myocarditis (pericardial effusion present in 2): myocardial edema by elevated T2 signal and myocardial injury by presence of nonischemic LGE. 12 athletes (46%) had LGE, of whom 8 (30.8%) had LGE without concomitant T2 elevation	N/A	Evaluation for myocarditis was based on the 2018 Lake Louise criteria.	**Myocarditis (T1 and T2 criteria)**: 15% (4/26); **suspected prior myocardial injury (LGE without T2 elevation)**: 30.8% (8/26)	14 (53.8%) asymptomatic; 12 (26.9%) mild; 2 athletes with cMRI findings of myocarditis were mildly symptomatic, while the other 2 were asymptomatic

**121**	Małek; **Poland**	To gain further insights into the relation between COVID-19 and cardiac involvement in professional athletes.	Elite athletes, N=26	Median age: 24 (range: 21-27); 19% male	**Cross-sectional study**: All athletes who test positive for COVID-19 underwent cMRI as soon as possible after infection. Additional tests performed on the day of cMRI were detailed physical examination, resting ECG, and laboratory tests, including whole blood count and markers of inflammation (C-reactive protein) and cardiac injury (cardiac troponin).	Median time from COVID-19 diagnosis to imaging: 32 (22-62)	**ECG**: No ST-T changes or Q waves identified. **cMRI**: No cMRI findings of acute myocarditis. Myocardial abnormalities were found in five (19%) athletes, including four athletes presenting borderline signs of isolated myocardial edema and one athlete showing nonischemic LGE with pleural and pericardial effusion. No correlation was observed between troponin and cMRI findings.	N/A	Evaluation for myocarditis was based on the 2018 Lake Louise criteria.	**Myocarditis (T1 and T2 criteria)**: 0% (0/26); **myocardial abnormalities (e.g. borderline signs of isolated myocardial edema, nonischemic LGE)**: 19.2% (5/26); **pericardial effusion**: 7.7% (2/26)	14 (54%) mild; 5 (19%) moderate; 6 (23%) asymptomatic; 1 (4%) severe/hospitalization; among those with myocardial abnormalities, 3 had mildly symptomatic COVID-19, whereas the other 2 had moderate symptoms
**122**	Brito; **US**	To explore the spectrum of cardiac abnormalities in student athletes who returned to university campus in July 2020 with uncomplicated COVID-19.	Student athletes, N=54	Median age: 19 (range: 19-21); >85% male	**Cohort study**: 54 patients with positive SARS-CoV-2 PCR or IgG antibody tests received cardiovascular screening, including an ECG and echocardiogram. Patients with symptomatic COVID-19 or asymptomatic patients with abnormal ECGs/echocardiograms underwent cMRI. Sequential cMRI was performed in 48 (89%) subjects; 2018 Lake Louise criteria used.	Median time from tests performed to imaging: 27 days (range: 22-33)	**ECG:** 1 inappropriate sinus tachycardia with ST-segment and T-wave changes. **Echocardiogram**: 2 with impaired LV function (abnormal GLS <16% or EF <50%); 8 with borderline RV dilation or dysfunction on echocardiography; small pericardial effusions (≥5 mm) were detected in 3 of these individuals. **cMRI**: No myocardial edema in all cases. 19 (40%) had late pericardial enhancement. 28 (58%) had pockets of pericardial effusion. Increased T1 (≥990 ms) was seen in 9 (19%) athletes, with 1 symptomatic individual showing late myocardial enhancement.	**Short-term follow-up**: 2/27 (7.4%) continued to have persistent symptoms and were advised to undergo cardiac rehabilitation program before returning to play.	1) Pericardial: defined as the presence of late enhancement with pericardial effusion on cMRI; 2) Myocardial: presence of any of the following criteria in isolation or in a combination: a) LVEF <50%, b) presence of regional wall motion abnormality, c) GLS <16%, and d) native T1 increase ≥990 ms; and 3) Myopericardial: a combination of items 1 and 2.	**Cardiac abnormalities (echocardiography and cMRI)**: 56.3% (27/48); myocardial involvement: 16.7% (8/48); pericardial involvement: 27.1% (13/48); myopericardial involvement: 12.5% (6/48); pericardial effusion: 58.3% (28/48); myocarditis (T1 and T2 criteria): 0% (0/48)	37 (77.1%) symptomatic; 11 (22.9%) asymptomatic
Sample size (N) >100											
**123**	Boehmer; **US**	To assess the association between COVID-19 and myocarditis using a large, U.S. hospital-based administrative database of health care encounters from >900 hospitals.	Patients with at least one inpatient or hospital-based outpatient encounter with myocarditis (excluding those vaccinated against COVID-19), N=5069; 16-	16-24-year-old cohort	**Cross-sectional study**: Adjusted risk differences were calculated as the difference between 1) the adjusted predicted risk for myocarditis (outcome) among patients with COVID-19 (exposed group) and 2) adjusted predicted risk for myocarditis among patients without COVID-19 (unexposed group); adjusted risk ratios (aRRs) were calculated as the ratio of the adjusted predicted risk among exposed to the	March 2020-February 2021	Among those aged 16-24 with myocarditis (511), 23.7% (n=121) had a history of COVID-19 (the majority occurred during the same month of infection). Among those with COVID-19, the risk % was 0.098 compared with 0.013 among those without COVID-19; the adjusted risk difference was 0.088 (95% CI: 0.061, 0.115); the aRR was 7.4 (95% CI: 5.5, 9.2).	N/A	A myocarditis ICD-10-CM code.	**Myocarditis (16–24-year-olds):** risk % of 0.098 among those with COVID-19 versus 0.013 among those without COVID-19	Patients with at least one inpatient or hospital-based outpatient encounter

			24-year-old age cohort, N=511		adjusted predicted risk among unexposed.						
**124**	Hendrickson; **US**	To determine the risk of COVID-19-related cardiovascular complications among athletes.	Student athletes, N=137	Median age: 20 (range: 18-27); 67.9% male	**Cohort study**: A 12-lead ECG, transthoracic echocardiogram, and conventional cardiac troponin I level were obtained from all athletes regardless of symptom history or illness severity. Anyone with an abnormal test result or clinical evaluation of concern was referred for cMRI. Clinical follow-up occurred through communication with university athletic staff.	Median time to evaluation after testing positive for COVID-19: 16 days (12-34)	**ECG**: No abnormal results. **Echocardiography**: no ventricular systolic or diastolic dysfunction, wall motion abnormalities, elevated right ventricular pressure estimates, or significant pericardial findings. Trace/small pericardial effusions in 4 (2.9%) athletes. **cMRI**: No athlete had abnormal findings. Trace pericardial effusions were observed in 2 athletes.	N/A	N/A	**Myocarditis (T2 weighted imaging and LGE)**: 0% (0/5); **trace/small pericardial effusions**: 2.9% (4/137)	75 (54.7%) mild; 37 (27%) moderate; 25 (18.2%) asymptomatic
**125**	Starekova; **US**	To describe the prevalence and severity of cMRI findings of myocarditis in a population of competitive student athletes recovering from COVID-19.	Student athletes, N=145	Mean age: 20 (range: 17-23); 74.5% male	**Cohort study**: An electronic health record search was performed at the University of Wisconsin to identify all competitive athletes recovering from COVID-19 who underwent gadolinium-enhanced cMRI. Serum markers of myocardial injury and inflammation, an electrocardiogram, transthoracic echocardiography, and relevant clinical data were obtained; 2018 Lake Louise criteria used.	Median time from a COVID-19 positive test to imaging: 15 days (range: 11-194)	**ECG**: In the first patient, nonspecific ST-T-wave abnormalities were detected 1 day before the MRI. **cMRI**: 2 (1.4%) patients had MRI findings consistent with myocarditis; of these, 1 had marked nonischemic late gadolinium enhancement and T2-weighted signal abnormalities over multiple segments, along with an abnormal serum troponin-I level; the second patient had 1-cm nonischemic mild late gadolinium enhancement and mild T2-weighted signal abnormalities, with normal laboratory values. A small non-specific focus of LGE was observed in the LV septum at the inferior RV insertion point in 38 of 145 patients.	**1-month follow-up**: T2-weighted signal abnormalities had improved in the first patient (and cardiac troponin normalized); T2-weighted signal abnormalities and LGE has resolved in the second patient.	Evaluation for myocarditis was based on the 2018 Lake Louise criteria.	**Myocarditis (T1 and T2 criteria)**: 1.4% (95% CI: 0.4%, 4.9%); 2/145; **non-specific focus of LGE in the left ventricular septum at the inferior right ventricular insertion point**: 26.2% (38/145)	71 (49.0%) mild; 40 (27.6%) moderate; 24 (16.6%) asymptomatic; of those with cMRI findings of myocarditis, 1 was initially asymptomatic (with mild dyspnea) and the other had mild to moderate symptoms lasting at least 3 days.
**126**	Erickson; **US**	To quantify the occurrence rate of abnormal electrocardiographic (ECG) findings and symptoms following COVID-19.	Student athletes, N=170	Mean age: ~19.5 (range: 18-25); 53.5% male	**Cohort study**: Athletes who previously tested positive for COVID-19 underwent general examinations and ECG screening prior to being medically cleared for return to sports following their COVID-19 diagnosis. Predetermined predictors (i.e. sex, symptom severity, body mass index) were grouped into categorical variables to examine differences of abnormal rates between categories.	Mean time from testing positive for COVID-19 and testing: 22.5 days (20.4-24.7)	**ECG**: 6 (3.5%) presented with abnormal or borderline ECG findings (e.g. ST depression, T-wave inversion, ST-T changes) and were referred to a cardiologist for further testing; no evidence that sex, symptom severity, and body mass index category were associated with a higher rate of abnormal ECG findings (P>.05). **Further diagnostic testing**: 2 cases of viral pericarditis among those who underwent further testing (n=16).	16 athletes were referred to cardiologists.	Two physicians evaluated all study participants for consistency in management and test interpretation using the international consensus criteria for ECG interpretation in athletes.	**Abnormal cardiac rhythms (via ECG)**: 3.5% (6/170); **pericarditis**: 1.2% (2/170)	Among those with abnormal cardiac rhythms, 8/16 (50%) reported mild symptoms and the other 8 (50%) reported moderate symptoms; the 2 athletes with pericarditis reported moderate symptoms

**127**	Moulson; **US**	To determine the prevalence and clinical implications of COVID-19 cardiac involvement in young competitive athletes.	Student athletes, N=3018 (42 colleges/universities)	Mean age: 20; 68% male	**Cohort study**: Evaluations included a clinical assessment and a range of cardiac testing as determined by the institution, including 1 or more of the following: 12-lead ECG, cardiac troponin assay, transthoracic echocardiogram (TTE), and cMRI. 'Triad' testing refers to the combination of ECG, troponin, and TTE. Athletes were either indicated for cMRI due to abnormal triad testing or symptom burden or as part of a primary cardiovascular screening protocol.	Median time from initial infection to cMRI: 33 days (18-63)	Cardiopulmonary symptoms during the acute illness or on return to exercise were reported by 13% of athletes. Abnormal triad testing possibly related to COVID-19-related cardiac involvement was detected by TTE (24 of 2556 [0.9%]), 12-lead ECG (21 of 2999 [0.7%]), and cardiac troponin testing (24 of 2719 [0.9%]); no athlete had abnormalities on all 3 triad tests. **cMRI**: 21/3018 (0.7% [95% CI: 0.4, 1.1]) cases of myocardial/pericardial involvement were identified: 6 of 198 (3.0% [95% CI: 1.1, 6.5]) athletes identified by primary screening cMRI, of whom 3 (1.5% [95% CI: 0.3, 4.4]) had definite or probable cardiac involvement; 15 of 2820 (0.5% [95% CI: 0.3, 0.9]) athletes identified by 1 or more elements of cardiac triad testing or moderate or greater symptoms followed by clinically indicated cMRI, of whom 12 (0.4% [95% CI: 0.2, 0.7]) had definite or probable cardiac involvement. For athletes in the clinically indicated cMRI cohort with at least 1 abnormal triad test (n=34), 6 (17.7% [95% CI: 6.8, 34.5]) had definite or probable cardiac involvement.	No adverse cardiac events (median follow-up period: 130 days (97-160) were reported in athletes with definite, probable, or possible cardiac involvement (n=21). Among the entire cohort of athletes with COVID-19 (n=3018; median follow-up period: 113 days [90-146]), there was 1 adverse cardiac event (a successfully resuscitated sudden cardiac arrest).	Cardiopulmonary symptoms were defined as chest pain, shortness of breath, palpitations, or exercise intolerance. ECGs were deemed normal or abnormal using international criteria for ECG interpretation in athletes. Evaluation for myocarditis was based on the 2018 Lake Louise criteria. For definitions of definite, probable, or possible cardiac involvement, refer to **Petek et al. Adverse cardiovascular events**: new clinically significant arrhythmias, clinical heart failure, or sudden cardiac arrest or death.	**Definite, probable, or possible cardiac involvement**: 0.7% (95% CI: 0.4, 1.1); 21/3018; **Definite or probable cardiac involvement:** 0.5% (15/3018); **myocarditis/pericarditis (definite cardiac involvement)**: 0.36% (11/3018); **definite, probable, or possible cardiac involvement (those who underwent cMRI)**: 6.6% (21/317)	33% asymptomatic; 29% mild
**128**	Petek; **US**	To assess the prevalence and clinical implications of persistent or exertional cardiopulmonary symptoms in young competitive athletes following COVID-19.	Student athletes, N=3597 (44 colleges/universities)	Mean age: 20; 66% male	**Cohort study**: Data from the Outcomes Registry for Cardiac Conditions in Athletes including US collegiate athletes recovering from COVID-19. Clinical characteristics, advanced diagnostic testing (e.g. cMRI) and COVID-19-associated sequelae were compared between athletes with persistent symptoms >3 weeks, exertional symptoms on return to exercise, and those without persistent or exertional symptoms. 'Triad' testing referred to the combination of 12-lead ECG, troponin, and transthoracic	Median time from initial infection to cMRI: 44 days (29-70)	Persistent symptoms >3 weeks were present in 44/3529 (1.2%) athletes with 2/3529 (0.06%) reporting symptoms >12 weeks; exertional cardiopulmonary symptoms were present in 137/3393 (4%) athletes. No athlete with persistent symptoms had abnormal triad testing (ECG 0/43, troponin 0/43, TTE 0/44). Triad testing was abnormal in 5 athletes with exertional symptoms, including 1/132 (0.8%) athlete who underwent ECG testing (diffuse T-wave inversions) and 4/117 (3.4%) athletes who underwent a TTE (2 LVEF <50%, 1 small pericardial effusion). **CV abnormalities**: 5 cases of probable/definite cardiac	N/A	Evaluation for myocarditis was based on the 2018 Lake Louise criteria. **Definite myocardial involvement** was defined as: (1) cMRI T1 abnormality or late gadolinium enhancement (LGE) plus T2 abnormality or (2) CMR T2 abnormality plus one or more supportive findings (left ventricular ejection fraction (LVEF) <45%, small or greater pericardial effusion, pericardial	**CV abnormalities (cardiac involvement, sinus tachycardia, POTS)**: 0.25% (9/3597); **probable or definite cardiac involvement**: entire cohort (unclear how many underwent cMRI): 0.14% (5/3597); athletes with exertional cardiopulmonary symptoms who underwent cMRI:	44 (1.2%) reported persistent symptoms >3 weeks from symptom onset

					echocardiogram (TTE). Advanced diagnostic testing was defined as any of the following (beyond triad testing): cMRI, cardiac stress testing, cardiopulmonary exercise testing, chest X-ray, coronary CT angiography, etc.		involvement (3 cases of pericardial involvement, 1 definite case of myopericardial involvement, and 1 probable case of myopericardial involvement), 2 cases of possible cardiac involvement, 2 cases of inappropriate sinus tachycardia, and 2 cases of postural orthostatic tachycardia syndrome (POTS). Of athletes with chest pain on return to exercise who underwent cMRI, 20.8% (5/24) had probable or definite cardiac involvement. In contrast, no athlete with exertional symptoms without chest pain who underwent cMRI (0/20) was diagnosed with probable or definite COVID-19-related cardiac involvement.		enhancement or troponin >99% upper limit of normal). **Probable myocardial involvement** was defined as: (1) cMRI T1 abnormality or presence of LGE plus one or more supportive finding (same as definite myocardial involvement). **Possible myocardial involvement** was defined as: (1) isolated cMRI T1 abnormality or presence of LGE. COVID-19 pericardial involvement was defined as a small or greater pericardial effusion or pericardial enhancement on cMRI. Any athlete meeting criteria for myocardial involvement of COVID-19 who also had pericardial involvement was labelled as definite, probable, or possible myopericardial involvement based on the definitions for myocardial involvement.	11.4% (5/44); athletes with exertional chest pain who underwent cMRI: 20.8% (5/24); **possible, probable, or definite cardiac involvement**: 0.19% (7/3597)	
Reviews/editorials											
**129**	Udelson; **US**	To summarize the most recent evidence published regarding the post-COVID-19 prevalence of cardiac abnormalities in athletes.	Student athletes, N=5846	N/A; 37-74% of college student athletes were male)	**Review/editorial**: N/A	Mean/median times to cMRI range from 15-33 days across studies with student samples	The prevalence of myocarditis detected via cMRI was 4/26, 2/145, 2/59, 15/317, 0/5, and 37/1597 from data collected on college athletes. The prevalence of myocarditis diagnosed in the full cohort was 4/26, 2/145, 2/59, 15/3018, 0/137, and 37/2461 from data collected on college athletes. The prevalence of non-myocarditis findings detected via cMRI was 8/26, 2/145, 1/59, 10/317, 0/5, and 81/1597 from	N/A	Evaluation for myocarditis was based on the 2018 Lake Louise criteria.	**Myocarditis** in those who underwent cMRI: 0-15%; Myocarditis in full cohort: 0-15%; non-myocarditis findings in those who underwent cMRI: 0-31%. Estimated prevalence of	16.6-54% of college student athlete cohorts were asymptomatic

							data collected on college athletes. The prevalence of signs of myocarditis on cMRI using the 2018 Lake Louise criteria is in the range of 1% to 3% in athletes following positive COVID-19 test results.			myocarditis on cMRI following positive COVID-19 test results: 1-3%	
**130**	van Hattum; **Netherlands**	To quantify pericardial/myocardial involvement and risks of sudden cardiac arrest/sudden cardiac death after COVID-19 in athletes who return to sports.	Student athletes, N=2326	Median age: 22 years; N/A	**Systematic review**: Conducted a search following PRISMA guidelines in academic databases through June 2, 2021. Studies were included when they: 1) contained clinical data on athletes recovered from COVID-19; (2) reported cardiovascular imaging by either cardiovascular magnetic resonance (cMRI) or echocardiography; (3) performed investigations after COVID-19, with emphasis on arrhythmia outcomes. Athletes were defined as individuals of young or adult age, either amateur or professional, who engage in regular exercise training and participate in official sports competitions.	Median time from symptom onset to imaging: 32 days (11-194)	Weighted means of diagnosed myocarditis were 2.1% in college/student athletes. The prevalence of myocarditis on echocardiography and/or cMRI was 0-15%, pericardial effusion 0-58%, and late gadolinium enhancement (LGE) 0-46% (markedly lower in studies with high-quality assessment scores [3-4%]). A single study reported reversibility of myocardial involvement in 40.7% of cases. No important arrhythmias or instances of sudden cardiac death were reported. Ten studies (n=4171) reporting post-recovery cardiac troponin found no clear relationship with cardiac abnormalities.	**113-day follow-up**: No important arrhythmias or instances of sudden cardiac death were reported.	Evaluations for myocarditis were based on Lake Louise criteria.	**Diagnosed myocarditis (college/student athletes)**: 2.1%	59% mild; 19% moderate; 0.2% severe; 22% asymptomatic

#	Authors (et al.); **country**	Study aim	Population of interest, N	Age (years); % male	Investigated	Methods	Pertinent results	Definitions	Estimates	Illness severity of participants
**Studies investigating MIS (n=2)**										
**131**	Belay; **US**	To describe the clinical characteristics and geographic and temporal distribution of the largest cohort of patients with MIS-C in the United States to date.	Patients suspected with MIS-C younger than 21 years, N=1733; 18-20-year-old cohort, n=55	18-20-year-old cohort; 67.3% male	Cardiovascular complications associated with MIS-C	**Cross-sectional study**: Patients diagnosed as having MIS-C were reported to the CDC by state, local, and territorial health departments using a standardized case report form, which included information on patient demographics, clinical findings, and laboratory test results. The patients were evaluated to determine if they met the CDC MIS-C case definition. Geographic and temporal distribution of MIS-C was compared	**18-20-year-old cohort**: Cardiovascular complications included hypotension in 52.7%, cardiac dysfunction in 41.5%, myocarditis in 30.9%, pericardial effusion in 26.8%, and coronary artery dilation or aneurysm in 14.6%. Patients aged 18 to 20 years had the highest proportion of myocarditis (30.9% in 18–20-year-olds versus 9.2-27.7% in 0-17-year-olds; p<0.001) and the highest proportion reporting preceding COVID-19–like illness (63%) 7 or more days before MIS-C onset, compared with younger cohorts. The proportion treated with intravenous immunoglobulin (29 [52.7%]) was lowest in patients aged 18 to 20 years. 58.2% (n=32) were admitted to the ICU, and 10.9% (n=6) died.	**MIS-C**: patients younger than 21 years hospitalized with fever, at least 2 organ system involvement, laboratory evidence of inflammation, and laboratory confirmation of COVID-19; patients were excluded if they had another plausible explanation for the illness. In addition, patients who met the following criteria were excluded: (1) respiratory manifestations in the absence of	**MIS incidence (18-20-year-olds)**: 0.4 per 100,000 infected; hypotension in 52.7%, cardiac dysfunction in 41.5%, myocarditis in 30.9%, pericardial effusion in 26.8%, and coronary artery dilation or aneurysm in 14.6%.	N/A

						with that of COVID-19 nationally, by region, and level of urbanicity by county. Clinical and laboratory findings and changes over time were described by age group and by presence or absence of preceding COVID-19.	The 18-20-year-old cohort had the lowest incidence of MIS at 0.4 per 100,000 infected (p<0.001).	cardiovascular, neurologic, or dermatologic manifestations; (2) respiratory symptoms and rash with no other symptoms and positive test results for COVID-19 by polymerase chain reaction (PCR); and (3) negative test results for COVID-19 by serology.		
**132**	Whitworth; **US**	To determine the incidence of thrombosis in children hospitalized with COVID-19 or MIS-C and evaluate associated risk factors.	Children hospitalized with COVID-19 or MIS-C, N=853; 16–21-year-olds that developed thrombosis, n=11	0 to <21 years (specifies 16–21-year-olds); 52.2% male	Thrombotic events	**Cohort study**: Patients aged 0 to <21 across 7 pediatric hospitals were classified into 1 of 3 groups for analysis: COVID-19, MIS-C, or asymptomatic COVID-19.	11 (55%) of the 20 patients who developed thrombosis were aged 16-21 (including 2 with a pulmonary embolism, 7 with a deep vein thrombosis, 1 with an intracardiac thrombosis, and 1 with an acute ischemic stroke), 36.4% (n=4) of which died. Risk factors for thrombosis included age 12 years and above, cancer, presence of a central venous catheter, and MIS-C; those aged 12 and older with MIS-C had the highest rate of thrombotic events at 19% (9/48).	COVID-19: a positive PCR and COVID-19 symptoms (fever, cough, diarrhea, loss of smell, headache, sore throat, or congestion); MIS-C: all 5 CDC criteria; MIS-C like: symptoms and laboratory findings highly suggestive of MIS-C in patients who were assessed and treated for MIS-C but did not fully meet the CDC criteria; and asymptomatic SARS-CoV-2: SARS-CoV-2+ PCR test without symptoms of COVID-19 (listed above) and admitted with alternative diagnoses.	**Rate of thrombotic events (aged 12 and older with MIS-C)**: 19% (9/48)	1 of 3 groups: COVID-19, MIS-C, or asymptomatic COVID-19.

#	Authors (et al.); **country**	Study aim	Sample size, N	Mean/median age (years)	Timeline of infection	Pertinent markers of CV function	Methods	Pertinent results
**Physiological studies investigating vascular function (n=5)**								
**133**	Ratchford; **US**	To determine if contracting COVID-19 may have prolonged effects on the systemic vasculature of otherwise	COVID-19 group: n=11 (4M/7F); control group: n=20 (5M/15F)	COVID-19 group: 20.1; control group: 23.0	3-4 weeks following a positive PCR test	**Brachial artery flow-mediated dilation [FMD]** (upper limb marker of vascular function); **femoral artery single passive leg movement [sPLM]** (lower limb marker of vascular function); **carotid-femoral pulse wave velocity [PWV]** (peripheral arterial stiffness)	All markers were measured using Doppler ultrasound.	**Brachial artery FMD**: lower in the COVID-19 group 2.71 ± 1.21% vs. 8.81 ± 2.96%; p<0.01; **Femoral artery sPLM**: lower in the COVID-19 group (-3 ± 91 mL vs. 118 ± 114 mL; p<0.01); **Carotid-femoral PWV**: higher in the COVID-19 group (5.83 ± 0.62 m/s vs. 5.17 ± 0.66 m/s; p<0.01)

		healthy young adults.						
**134**	Szeghy; **US**	To investigate whether carotid stiffness, carotid intima–media thickness and the aortic augmentation index are altered in young adults 3-4 weeks after contraction of SARS-CoV-2 compared with young healthy adults.	COVID-19 group: n=15 (6M/9F); control group: n=15 (5M/10F)	COVID-19 group: 20; control group: 23	25 +/-5 days after symptom onset and 24 +/- 6 days after a positive PCR test	**Young's modulus** (carotid artery stiffness); **intima-media thickness [cIMT]; aortic augmentation index [AIx]** (marker of ventricular-vasculature coupling); **systolic pressure-time index [SPTI]** (index of myocardial oxygen demand)	Carotid pulse wave was recorded using a SphygmoCor CPv device. All other values were measured using Doppler ultrasound.	**Young's modulus**: greater in SARS-CoV-2 group (0.59 ± 0.24 kPa vs. 0.40 ± 0.12 kPa; p=0.01); **Aortic AIx**: greater in SARS-CoV-2 group 12.7 ± 9.1% vs. 3.3 ± 12.6%; p=0.03); **SPTI**: greater in SARS-CoV-2 group (2429 ± 368 vs. 2099 ± 447; p=0.04)
**135**	Nandadeva; **US**	To investigate the persistent effects of COVID-19 on vascular function in otherwise healthy young adults.	COVID-19 group: n=16 (8 symptomatic [SYM]/8 asymptomatic [ASYM]); control group: n=12	COVID-19 group: 23; control group: 23	At least 4 weeks past a COVID-19 diagnosis	**Brachial artery flow-mediated dilation [FMD]**; **peak blood velocity following cuff release** (microvascular function); **carotid-femoral PWV** (marker of ventral arterial stiffness); **Alx** (marker of central arterial stiffness)	FMD was measured using Doppler ultrasound; central arterial stiffness was assessed by carotid-femoral pulse wave velocity and pulse wave analysis.	**FMD**: lower in SYM (3.8 ± 0.6%) compared to ASYM (6.8 ± 0.9%; p=0.007) and control (6.8 ± 0.6%; p=0.003); **Peak blood velocity following cuff release**: lower in SYM (47 ± 8 cm/s) compared with ASYM (64 ± 19 cm/s; p=0.025) and control (61 ± 14 cm/s; p=0.036)
**136**	Stute (2021); **US**	To investigate autonomic (dys)function and haemodynamics in otherwise healthy young adults recently infected with SARS-CoV-2.	COVID-19 group: n=16 (8M/8F); control group: n=14 (5M/9F)	COVID-19 group: 20.4; control group: 21.4	3-8 weeks after a positive PCR test	**Muscle sympathetic nerve activity [MSNA]** (associated with increased arterial stiffness); **resting heart rate**; **systolic blood pressure [SBP]**; **diastolic blood pressure [DBP]**	Multiunit MSNA was assessed using the microneurographic technique with a recording electrode and a reference electrode.	**Resting MSNA burst frequency**: higher in COVID-19 group (18.2 ± 6.6 bursts/min) compared to control (12.7 ± 3.4 bursts/min; p=0.02) **Resting MSNA total activity:** higher in COVID-19 group (285 ± 101 a.u./min) compared to control (159 ± 46 a.u./min; p=0.001) **Resting heart rate**: no difference between COVID-19 group (63 ± 9 beats/min) compared to control (65 ± 9 beats/min; p=0.435) **SBP**: no difference between COVID-19 group **(**132 ± 10 mmHg) compared to control (129 ± 13 mmHg; p=0.399) **DBP**: no difference between COVID-19 group (78 ± 4 mmHg) compared to control (75 ± 8 mmHg; p=0.157)
**137**	Stute (2022); **US**	To investigate whether central and peripheral hemodynamics during handgrip exercise different in young adults 3-4 weeks following COVID-19 compared with young healthy adults.	COVID-19 group: n=13 (8M/5F); control group: n=13 (8M/5F)	COVID-19 group: 21; control group: 27	25 days after a positive PCR test	**Rate pressure product [RPP]** (indication of myocardial oxygen consumption); **FMD**; **brachial artery blood flow, vascular conductance**; **heart rate**; **stroke volume**; **cardiac output**	Heart rate, stroke volume, and cardiac output were determined using beat-to-beat photoplethysmography; other variables were measured using Doppler ultrasound.	**RPP**: higher in COVID-19 group at 45% maximal voluntary contraction (MVC) compared to control (12975 ± 2686 mmHg bpm vs. 9727 ± 1928 mmHg bpm; p<0.001); **Brachial artery blood flow**: lower in COVID-19 group at 45% MVC (386.3 ± 132.5 mL/min vs. 507.4 ± 109.9 mL/min; p=0.002); **Brachial artery vascular conductance**: lower in COVID-19 group at 45% MVC (3.46 ± 1.10 mL/min/mmHg vs. 4.74 ± 1.02 mL/min/mmHg; p<0.001)

#	Authors (et al.); **country**	Study aim	Population of interest, N	Age (years); % male	Investigated	Methods	Pertinent results	Definitions	Estimates	Illness severity of participants
**Studies investigating CHD (n=3)**										
**138**	Lewis; **US**	To assess the impact and predictors of COVID-19 infection and severity in a cohort of patients with CHD.	Patients with CHD and COVID-19, N=53; 21–25-year-olds, n=4	Median age: 34 (specifies 21-25-year-olds); 58% male	CHD and severity of COVID-19	**Retrospective review**: A retrospective review of all individuals with CHD diagnosed with COVID-19 was performed at Columbia University Irving Medical Center between March 1, 2020, and July 1, 2020. The primary end point was moderate/severe response to COVID-19 infection. Echocardiographic data were obtained via historical review of transthoracic echocardiograms read by cardiologists who were board-certified in congenital heart disease.	History of a genetic syndrome and an advanced physiological stage of CHD (class C or D) were significantly associated with moderate-to-severe COVID-19 infection (odds ratios of 35.82 and 19.38, respectively). In addition, individuals with ventricular dysfunction were more likely to have a longer duration of symptoms. Four emerging adults aged 21-25 were included in the cohort; these patients were described to have moderate-to-severe COVID-19 infection, requiring hospitalization (n=3), supplemental oxygen (n=3), and a tracheostomy (n=1).	Moderate/severe response to COVID-19 infection defined as: (1) death during COVID-19 infection; or (2) need for hospitalization and/or respiratory support secondary to COVID-19 infection.	**Moderate/severe COVID-19 (CHD patients)**: 17% (9/53); **COVID-19-associated death**: 6% (3/53)	9 (17%) moderate/severe; 3 (6%) died
**139**	Broberg; **US**	To define the impact of COVID-19 in adults with CHD and identify risk factors associated with adverse outcomes.	Adults with CHD, N=1044; 18-27-year-old cohort, n=356	Mean age: 35.1 (range: 18-86) [specifies 18–27-year-olds]; 49% male	CHD and adverse outcomes of COVID-19	**Cohort study**: Adults (aged ≥18) with CHD and with confirmed or clinically suspected COVID-19 were included from CHD centers worldwide. Data collection included anatomic diagnosis and subsequent interventions, comorbidities, medications, echocardiographic findings, presenting symptoms, course of illness, and outcomes. Predictors of death or severe infection were determined.	There were 24 COVID-related deaths (case fatality rate: 2.3%; 95% CI: 1.4, 3.2). Factors associated with death included male sex, diabetes, cyanosis, pulmonary hypertension, renal insufficiency, and previous hospital admission for heart failure. A worse physiological stage was associated with mortality (p=0.001), whereas anatomic complexity or defect group were not. **18-27-year-old cohort**: mortality in 7/356 (2%) patients; 0-0.7% among those with physiological Stage A (0/87) and B (1/136); 3.5-11.1% among those with physiological stage C (4/115) or D (2/18).	A hierarchical classification system was used to categorize patients by their single most complex defect type, such that each patient was included in only one defect category (e.g. patients identified as having Eisenmenger physiology, with both cyanosis and pulmonary hypertension in the setting of a shunt, were designated as such, and removed from their respective anatomic defect category).	**Case fatality rate (18–27-year-olds with CHD)**: total cohort: 7/356 (2%); patients with physiological Stage A and B: 0-0.7%; physiological Stage C and D: 3.5-11.1%	60 (6%) asymptomatic; 179 (17%) hospitalized
**140**	Fisher; **US**	To describe outcomes of acute COVID-19 in pediatric and young adult	Pediatric and young adult patients with	Mean age: 7.7 (range: 0-22) [specifies	Underlying cardiac disease and COVID-related hospitalization	**Retrospective review**: A retrospective single-institution review was conducted of patients with known cardiac	Hospitalizations for COVID-19 were rare among children and young adults with underlying cardiac disease. Noncardiac comorbidities were associated with an increased	Please refer to the article for categorizations of CHD and acquired heart disease.	N/A	19 (47.6%) asymptomatic; 16 (38.1%) mild; 6 (14.3%) hospitalized

#	Authors (et al.); **country**	Study aim	Population of interest, N	Age (years); % male	Investigated	Methods	Pertinent results	Definitions	Estimates	Illness severity of participants
		patients with underlying cardiac disease and evaluate the association between cardiac risk factors and hospitalization.	underlying cardiac disease, N=42; 18-22-year-old cohort, n=6	those aged ≥18]; 57.1% male		disease and COVID-19 from March 1, 2020 to November 30, 2020. Extracardiac comorbidities and cardiac risk factors were compared between those admitted for COVID-19 and the rest of the cohort using univariate analysis.	risk of hospitalization (p=0.02), particularly chronic pulmonary disease (p=0.01) and baseline supplemental oxygen requirement (p=0.007). Extracardiac comorbidities like pulmonary disease were associated with increased risk of hospitalization, while cardiac risk factors were not.			
**Studies investigating risk factors for severe COVID-19, related cardiovascular complications, and mortality (n=7)**										
**141**	Kibbey; **US**	To document the effects of the COVID-19 pandemic on the mental health of undergraduate students in a heavily impacted area in the United States.	Undergraduate students, N=641	Mean age: 20.1; 27.3% male	Heart conditions (aggregated)	**Cross-sectional study**: Cross-sectional, self-report data on psychological distress and COVID-19 exposure were collected from a racially and ethnically diverse sample of undergraduates between April 7, 2020 and May 9, 2020.	1.9% (12/641) of college students reported the lifetime presence of a heart condition	No definition provided for 'heart condition'.	**Heart conditions**: 1.9% (12/641)	48% with at least 3 symptoms
**142**	Majumdar; **India**	To determine changes in various parameters (e.g. mental and physical health, depressive status, sleep quality) before and after a COVID-19 lockdown in office workers and undergraduate students.	Undergraduate students, N=325	Mean age (undergraduate students): 22.1; 39.1% male	Hypertension/high blood pressure	**Cross-sectional study**: An online questionnaire was posted on April 14, 2020 during a COVID-19 lockdown in India. The respondents comprised two groups: (i) individuals engaged in the corporate sector, performing a '9-5′ work while confined at home; and (ii) university undergraduate or postgraduate students. Diagnosed hypertension/high blood pressure was self-reported.	The prevalence of hypertension/high blood pressure among university students remained at ~1% after the COVID-19 lockdown.	Diagnosed hypertension/high blood pressure was self-reported.	**Hypertension/high blood pressure (undergraduate students, before and after a COVID-19 lockdown)**: ~1%	N/A
**143**	Altonen; **US**	To review 395 young adults aged 18-35 admitted for COVID-19 to one of the eleven hospitals in New	Young adults admitted for COVID-19, N=395; 18-23-year-old cohort, n=40	Mean age: 29.2; 66.8% male	Hypertension; cardiac comorbidities	**Retrospective review**: Demographics, comorbidities, clinical course, outcomes, and characteristics linked to hospitalization were analyzed including	Cardiac comorbidities (Wilcoxon p=0.015) and hypertension (Wilcoxon p=0.03) were significantly associated with increased mortality. Among those aged 18-23, two were diagnosed with hypertension (0.5%); these	A list of cardiac comorbidities (e.g. heart failure, cardiac murmurs) is provided in the article.	**Hypertension (hospitalized 18–23-year-olds)**: 5% (2/40)	All patients were admitted to the hospital.

		York City public health system.				temporal survival analysis.	individuals recovered from COVID-19.			
**144**	Adams; **US**	To examine young adults' medical vulnerability to severe COVID-19 illness.	Young adults aged 18-25, N=8405	Ages 18-25; 50.4% male	Heart conditions (aggregated)	**Cross-sectional study**: A young adult subsample (aged 18-25) was developed from the National Health Interview Survey (2016-2018), a nationally representative data set. The medical vulnerability measure (yes vs. no) was developed, guided by the Centers for Disease Control and Prevention (CDC) medical indicators. The estimates of medical vulnerability were developed for the full sample, the nonsmoking sample, and the individual risk indicators.	0.5% (34/8405) participants reported heart conditions, one of the medical vulnerabilities for severe COVID-19 illness established by the CDC.	Heart condition: coronary heart disease, angina, and heart attack.	**Heart conditions (18–25-year-olds)**: 0.5% (34/8405)	N/A
**145**	Fathi; **Iran**	To develop a prognostic model for predicting the risk of two-week mortality in patients with COVID-19.	Adults with COVID-19 admitted to healthcare centres, N=57705; 15-24-year-old cohort, n=1049	Mean age: 56.7 (specifies those aged 15-24); 53.1% male	Hypertension; cardiovascular disease; COVID-19-related mortality	**Cohort study**: The study registration process was online using a single electronic form. Most of the data were collected through patients' interviews early in admission and in reviewing their medical records. Data from the capital province (Tehran) were used for testing, and the data from all other provinces were used for model development. The selected variables were incorporated into three distinct models for the age categories using logistic regression.	50 (4.8%) participants aged 15-24 who died within 2 weeks; hypertension was significantly associated with mortality (OR: 54.27; 95% CI: 19.86, 168.19).	N/A	**2-week case fatality rate (admitted 15-24-year-olds)**: 4.8% (50/1049)	All participants were inpatients.
**146**	Richardson; **US**	To characterize young adult patients hospitalized with COVID-19 and identify predictors	Hospitalized patients aged 18-39, N=1013; 18-24-year-old cohort, n=119	Median age: 33 (range: 28-36) [specifies those aged 18-24]; 48% male	Heart failure	**Cohort study**: Data from 12 acute care hospitals in the New York City area. Patients aged 18–39 hospitalized with confirmed COVID-19	4 patients (3.4%) aged 18-24 died; among those who died or required invasive mechanical ventilation (n=7), all were obese, and five of these patients had additional comorbidities, including Down	N/A	**Case fatality rate (hospitalized 18–24-year-olds)**: 3.4% (4/119)	18–24-year-olds: 115 (96.6%) remained alive; 4 (3.4%) died

		of survival at 30 days.				between March 1, 2020 and April 27, 2020 were included in the study. Demographic, clinical, and outcome data were extracted from electronic health record reports.	syndrome and congestive heart failure.			
**147**	Sandoval; **US**	To investigate demographic and clinical risk factors for severe disease and readmission among young adults aged 18-29 who were diagnosed with COVID-19 at a hospital encounter in Houston, Texas, US.	Young adults aged 18-29, N=1853	Mean age: 24 (range: 21-27); 38% male	Composite disease outcome (includes cardiovascular events)	**Retrospective review**: The study included all consecutive patients aged 18-29 diagnosed at a hospital encounter with COVID-19 between March 1, 2020 and December 7, 2020 within Houston Methodist affiliated hospitals. Demographic, geographic, and clinical data were retrieved from the Houston Methodist COVID-19 Surveillance and Outcomes Registry. Logistic regression was performed to determine the risk factors for outcomes among COVID-19 cases (composite disease outcomes, pneumonia, and returning to the hospital within 30 days of discharge).	The cohort was relatively healthy: 78% of patients scored 0 on the Charlson Comorbidity Index Score, though 1,252 (68%) were overweight (25–30 kg/m2) or obese (≥30 kg/m2). Of the 148 diagnosed with at least composite disease outcomes, 2 had an MI, 8 had a pulmonary embolism, 3 went into cardiac arrest, 4 had a cerebrovascular accident, and 9 had a thrombosis. In multivariable logistic regression analysis, increasing age (adjusted odds ratio [aOR] 1.1; 95% CI: 1.1, 1.2), male sex (aOR 1.8; 95% CI: 1.2, 2.7), Hispanic ethnicity (aOR 1.9; 95% CI: 1.2, 3.1), obesity (>40 kg/m2) (aOR 3.8; 95% CI: 2.4, 6.0), asthma, myocardial infarction history, congestive heart failure, cerebrovascular disease, diabetes, and solid organ transplant history were significantly associated with a composite disease outcome within 30 days of initial encounter (Table 4). Congestive heart failure (aOR 6.0; 95% CI: 1.5, 25.1) and cerebrovascular disease (aOR 4.9; 95% CI: 1.7, 14.7), were significant predictors of severe disease diagnoses within 30 days. Obesity (aOR 1.7: 95% CI: 1.0, 2.9) and myocardial infarction history (aOR 6.2: 95% CI: 1.7, 23.3) were significantly predictors of 30-day readmission.	Composite disease outcomes (within 30 days of initial encounter): sepsis, myocardial infarction, cerebrovascular event, cardiac arrest, pulmonary embolism, thrombosis, acute respiratory distress syndrome (ARDS), acute respiratory failure (ARF), pneumothorax, gastrointestinal bleed, acute kidney injury, hypoxemia, shock, or systemic inflammatory response syndrome (SIRS).	**Case fatality rate (hospitalized 18-29-year-olds)**: 0.16% (3/1853)	1547 (83.5%) were discharged home; 40 (2.2%) remain hospitalized; 263 (14.2%) returned to hospital; 3 (0.16%) died

Supplemental file abbreviations: cMRI, cardiac magnetic resonance imaging; CV, cardiovascular; CVD, cardiovascular disease; CHD, congenital heart disease; COVID-19, coronavirus disease 2019; ECG, electrocardiogram; IgG, immunoglobulin G; IVIG, intravenous immunoglobulin; LGE, late gadolinium enhancement; MIS-C/A, multisystem inflammatory syndrome in children/adults; N/A, not applicable/available; PCR, polymerase chain reaction; SARS-CoV-2, severe acute respiratory syndrome coronavirus 2; US, United States; UK, United Kingdom.
